# On the Configurational Stability of Chiral Heteroatom-Substituted [D_1_]Methylpalladium Complexes as Intermediates of Stille and Suzuki–Miyaura Cross-Coupling Reactions

**DOI:** 10.1002/ejoc.201300439

**Published:** 2013-06-27

**Authors:** Petra Malova Krizkova, Friedrich Hammerschmidt

**Affiliations:** [a] Institute of Organic Chemistry, University of Vienna, Währingerstrasse 38, 1090 ViennaAustria

**Keywords:** Palladium, Isotopic labeling, Enantioselectivity, Cross-coupling, Chi­rality, Reaction mechanisms

## Abstract

Enantiomerically pure (*S*)-tributylstannyl[D_1_]methanol and (*R*)- and (*S*)-tributylstannyl[D_1_]methyl benzoates were Stille-coupled with bromobenzene and benzoyl chloride in 1,4-dioxane and toluene using [(Ph_3_P)_4_Pd] or [(Ph_3_P)_2_PdCl_2_] either alone or in combination with CuCN as cocatalyst at temperatures up to 80 °C. The products were found to be enantiomerically pure. (*R*)- and (*S*)-*N*-(tributylstannyl[D_1_]methyl)phthalimides gave enantiomerically pure products with benzoyl chloride, but with bromobenzene protected phenyl[D_1_]methylamines gave products of only 52–69 % *ee* depending on the solvent used. Tributyl(thio[D_1_]methyl)stannanes could not be Stille-coupled with benzoyl chloride or with bromobenzene. Similarly, dimethyl phenyl[D_1_]methylboronate underwent a Suzuki–Miyaura coupling with bromobenzene to give phenyl[D_1_]methylsilane with 99 % *ee*. All couplings followed a retentive course and, except in one case, the intermediate [XCHDPdL*_n_*] complexes were found to be microscopically configurationally stable.

## Introduction

**Figure 1:**
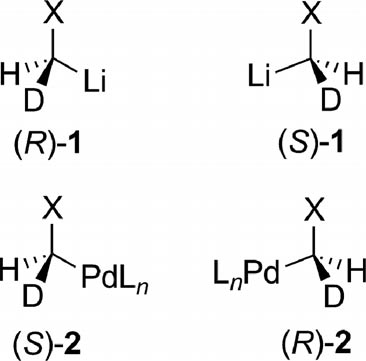
Chiral [D_1_]methyllithium compounds 1 and [D_1_]methylpalladium compounds 2.

Chiral, α-heteroatom-substituted organolithium compounds that are configurationally stable (at low temperatures) are valuable reagents for preparative, synthetic, stereochemical, mechanistic, and theoretical reasons.[1] Still and Sreekumar were the first to demonstrate that α-heteroatom-substituted alkyllithium compounds are configurationally stable at low temperature. They found that α-alkoxyalkyllithium compounds can be generated easily from the corresponding stannanes and that they react with electrophiles.[2] This finding sparked a broad interest in α-heteroatom-substituted alkyllithium compounds. Just to name three, the groups of Hoppe,[3] Beak,[4] and Hoffmann,[5] pioneered this field and developed methods to prepare these organolithium compounds enantioselectively and to determine their configurational stability. Although many theoretical calculations were performed on heteroatom-substituted methyllithium compounds as analogues for alkyllithium compounds,[6] they were not evaluated experimentally, because they were not accessible until recently. We concentrated on these most simple cases, the chiral α-heteroatom-substituted methyllithium compounds[7] **1**, and studied their microscopic (on the time scale of a rearrangement) and macroscopic (addition to benzaldehyde after aging) configurational stability (Figure 1). The substituent X comprised oxygen-,[8] sulfur-,[9] and nitrogen-containing[10] moieties and chloride[11] and bromide.[9] In summary, chiral methyllithium compounds with oxygen as heteroatom were configurationally stable at –78 °C or even higher temperatures, whereas nitrogen-containing chiral methyllithium compounds were only partly stable even at –95 °C. Chiral chloro- and bromo[D_1_]methyllithium are chemically very labile, but configurationally stable.[11] This paper focuses on intermediates of structural type **2** in palladium-catalyzed cross-coupling reactions.

## Results and Discussion

**Scheme 1:**
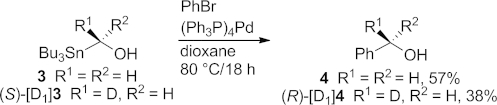
Stille coupling of (tributylstannyl)methanols and PhBr with Pd^0^L*_n_*.

To widen the scope for the preparation of compounds containing an attached chiral XCHD substituent, we decided to use the Stille cross-coupling[12] to transfer chiral [D_1_]methyl groups. The required stannanes were either known or could be obtained from (*R*)- and (*S*)-tributylstannyl[D_1_]methanol[8] in 99 % *ee*. To use this transformation in more complex settings, the overall stereochemistry was first addressed with simple substrates. Because the Stille coupling of (tributylstannyl)methanol and bromobenzene with tetrakis(triphenylphosphane)palladium had previously been studied[13] and the conditions had been optimized, we repeated this experiment with the unlabeled and subsequently with the labeled species (Scheme 1).

**Figure 2:**
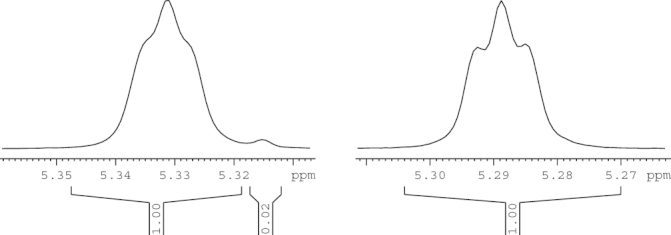
^1^H NMR signals of the CHD groups of the (*R*)-Mosher esters of (*R*)-phenyl[D_1_]methanol (left) and authentic (*S*)-phenyl[D_1_]methanol (right).

**Scheme 2:**
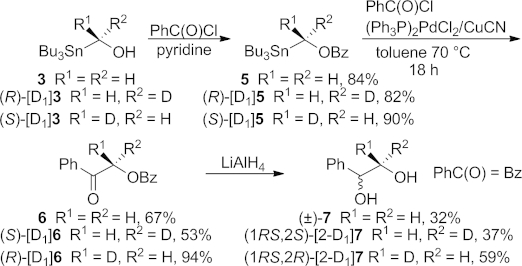
Stille cross-coupling of (tributylstannyl)methyl benzoates 5 with PhC(O)Cl.

As we were primarily interested in the stereochemical outcome of the transfer of the chiral methyl group, the yield of 38% for (*R*)-[D_1_]**4** (57% for **4**), although lower than the yield previously[13] reported for **4** (83%), was not of major concern. When starting from stannylmethanol (*S*)-[D_1_]**3**, the isolated deuterated benzyl alcohol and an authentic sample of the (*S*) enantiomer[14] were converted into the (*R*)-Mosher esters[15] using Mosher chloride [(*S*)-MTPACl]/pyridine. They were investigated by ^1^H NMR spectroscopy (400 MHz, CDCl_3_) to determine their *de*, which corresponded to the *ee* of the underlying [D_1_]**4**, and the configuration of the alcohol obtained by coupling. The ^1^H NMR spectrum of the latter displayed a broadened singlet at *δ* = 5.33 ppm, whereas the ^1^H NMR spectrum of the (*R*)-Mosher ester of an authentic sample of (*S*)-phenyl[D_1_]methanol displayed the signal for the CHD group at *δ* = 5.29 ppm (Figure 2). As we started from stannylmethanol (*S*)-[D_1_]**3** and obtained deuterated phenylmethanol (*R*)-[D_1_]**4** with 99 % *ee*, the Stille coupling clearly followed a net retentive course. On the basis of the accepted mechanism,[16] the chiral complex [(HOCHD)PhPdL*_n_*] must therefore be configurationally stable at 80 °C from the formation of [PhPdL*_n_*] and stannane to the reductive elimination of phenylmethanol with regeneration of Pd^0^L*_n_*.

(Tributylstannyl)methyl benzoate (**5**) and both labeled isotopomers and benzoyl chloride were coupled next (Scheme 2).

Falck et al. found, that various acetates and benzoates of α-(tributylstannyl) alcohols can be cross-coupled with acyl chlorides with retention of configuration, cocatalyzed by Cu^I^ salts.[17] However, Labadie and Stille reported that phenyl-, alkyl-, and vinylstannanes could be coupled with benzoyl chloride without using Cu^I^CN as cocatalyst.[18] The benzoates were easily accessible and were cross-coupled by the procedure reported in the literature.[17] To determine the configuration and *ee* at C-1 of esters **6** and (*S*)- and (*R*)-[D_1_]**6**, they were reduced with LiAlH_4_ to diol (±)-**7** and mixtures of diastereomeric diols (1*RS*,2*R*)- and (1*RS*,2*S*)-[2-D_1_]**7**, respectively. The latter two mixtures were converted into mixtures of (*R*)-Mosher esters, having (*S*) and (*R*) configuration at C-2, respectively (C-2 of the diol corresponds to C-1 of the benzoylated hydroxy ketone [D_1_]**6**).[8a] For clarity, it is more convenient here and in similar cases to give the enantiomeric excess for each chiral center of diol [2-D_1_]**7** individually (“*ee*”). Because the reduction of [D_1_]**6** is not enantioselective, the diol will be racemic at C-1 and therefore the “*ee*” at C-1 will be zero. The “*ee*” at C-2 of both diols [D_1_]**7** was higher than 99 %, deduced from the “*de*” of more than 99 % at C-2 as determined by ^1^H NMR spectroscopic analysis. Therefore, the Stille cross-coupling occurred with net retention of configuration, and the intermediate chiral methylpalladium complex [{PhC(O)}Pd{CHDOC(O)Ph}L*_n_*] is microscopically configurationally stable in toluene at 70 °C.

**Scheme 3:**
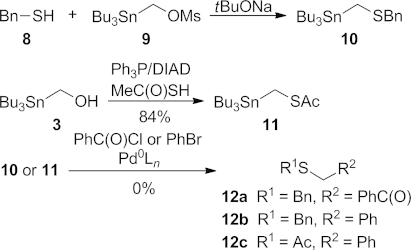
Preparation of starting materials 10 and 11 and attempted Stille coupling.

To see whether sulfur-substituted chiral methyl groups can be transferred by the Stille reaction, the respective unlabeled isomers of **10** and **11** were prepared and tested first (Scheme 3). The sodium salt of benzylmercaptane (**8**) was alkylated with mesylate[8b] **9** to yield sulfide **10**.[9] The thioacetate **11** was obtained in 84 % yield by the Mitsunobu reaction[19] of stannylmethanol **3** with thioacetic acid. We attempted to cross-couple these two stannanes with either benzoyl chloride or bromobenzene with 5 mol-% [(Ph_3_P)_2_PdCl_2_] either with or without CuCN (10 mol-%), [(Ph_3_P)_4_Pd] (5 mol-%), or [(*t*Bu_3_P)_2_Pd] (3 mol-%)[20] in toluene at temperatures up to 75 °C or dioxane up to 100 °C. No coupling product could be detected, although **12a**[21] and **12b**[22] were prepared as reference samples for unequivocal detection of the desired products by TLC and by ^1^H NMR spectroscopy. A part of the starting material could be recovered. When the stannane was activated[20] with 2.2 equiv. of CsF for transfer of the benzylthiomethyl group to PhBr, and [(*t*Bu_3_P)_2_Pd] was used as catalyst in dioxane as solvent, the starting material was consumed after 25 h at 90 °C. However, the desired product could not be detected in the crude material. On the other hand, a compound derived from the stannane by loss of two butyl groups, reminiscent of findings by Stille[18] and Falk,[17] was found.

**Scheme 4:**
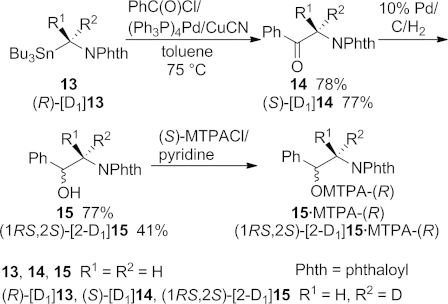
Stille reaction of *N*-[(tributylstannyl)methyl]phthalimides 13 and (*R*)-[D_1_]13.

Finally, we studied the transfer of the *N*-protected aminomethyl and chiral amino[D_1_]methyl groups from known stannanes[10] **13** and (*R*)-[D_1_]**13** with benzoyl chloride or bromobenzene as our standard organic halides. The cross-coupling in toluene with [(Ph_3_P)_4_Pd] and benzoyl chloride was a smooth reaction, giving *N*-protected amino ketones **14** and an impurity, which could be removed by flash chromatography, assuming (preferential) transfer of the aminomethyl group with retention of configuration (Scheme 4). To evaluate the *ee* at the stereogenic center, the sluggish catalytic reduction of a reference sample of **14** with a large amount of catalyst was first performed a few times. Although the reproducibility of the yield was low, sufficient amounts of alcohols **15** and (1*RS*,2*S*)-[2-D_1_]**15** were obtained for derivatization with (*S*)-Mosher chloride. The *ee* was found to be ≥ 98 % (^1^H NMR, 600 MHz, CDCl_3_). In analogy to the transfer of the chiral oxy[D_1_]methyl group by the Stille reaction, we assume net retention of configuration also occurs for the aminomethyl group.

**Scheme 5:**
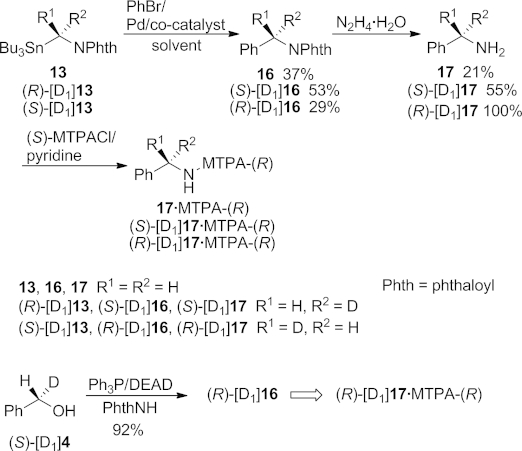
Stille reaction of *N*-[(tributylstannyl)methyl]phthalimides 13 and bromobenzene.

**Figure 3:**
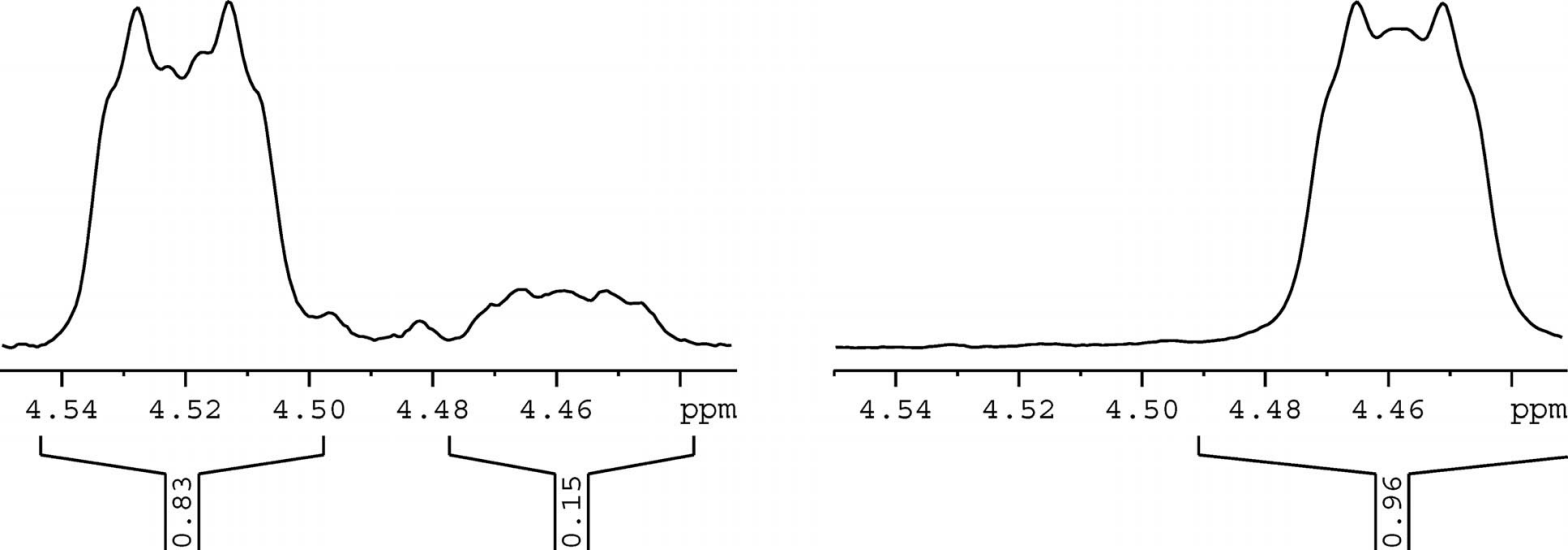
Signals of the CHD group in the ^1^H NMR spectra (400 MHz) of (*R*)-Mosher amides derived from (*S*)-[D_1_]16 (left, 69 % ee) and authentic (*R*)-[D_1_]16 (right, 99 % ee).

Finally, benzoyl chloride was replaced by bromobenzene in the Stille coupling (Scheme 5). Despite optimization of the reaction conditions in the unlabeled series by using various combinations of a Pd catalyst either alone or with CuCN as cocatalyst and toluene or dioxane as solvent, the yield was 37 % at best (Table 1). Rather high quantities of Pd catalyst (8 mol-%) and CuCN (16 mol-%) were required, and the solvent did not seem to influence the yield (Entries 3 and 4). The use of [{(*t*Bu)_3_P}_2_Pd] as catalyst either alone or in combination with CsF did not give any coupling product. The chiral stannanes (*R*)- and (*S*)-[D_1_]**13** were coupled under identical conditions, except that dioxane and toluene were used, respectively (Entries 5 and 6). Hydrazinolysis of phthalimides **16** gave benzylamines **17**, which were derivatized with (*S*)-MTPACl to furnish amides that were suitable for the determination of the *ee* by ^1^H NMR spectroscopy. An authentic sample of (*R*)-phenyl[D_1_]methylamine [(*R*)-[D_1_]**16**] was prepared from (*S*)-phenyl[D_1_]methanol and converted into the (*R*)-Mosher amide. The ^1^H NMR spectra (400 MHz) show that stannanes (*R*)- and (*S*)-[D_1_]**13** gave labeled *N*-benzylphthalimides of the same configuration, implying a net retentive course for the coupling. However, the enantiomeric excesses were 52 % (dioxane as solvent) and 69 % (toluene as solvent), respectively (Figure 3). Suginome et al. performed Suzuki–Miyaura couplings of enantioenriched [α-(acylamino)benzyl]boronic esters (acyl = acetyl, propionyl, benzoyl, pivaloyl) with aryl chlorides and bromides.[23] However, they found that the formation of the C–C bond was highly stereospecific and followed an invertive course. This was attributed to a transition state for transmetalation with a strong intramolecular coordination of the carbonyl group to the boron atom, which is not possible in stannanes **13**.

**Table 1:** Stille reaction of (aminomethyl)stannanes 13 with bromobenzene.

Entry	Stannane	Catalyst (mol-%)	Solvent	*T*	*t*	Yield
		Cocatalyst (mol-%)		[°C]	[h]	[%]
1	**13**	[(Ph_3_P)_2_PdCl_2_] (4)	toluene	80	20	13
		CuCN (8)				
2	**13**	[(Ph_3_P)_4_Pd] (5)	dioxane	80	20	14
3	**13**	[(Ph_3_P)_2_PdCl_2_] (8)	toluene	110	6	37
		CuCN (16)				
4	**13**	[(Ph_3_P)_2_PdCl_2_] (8)	dioxane	100	6	37
		CuCN (16)				
5	(*R*)-[D_1_]**13**	[(Ph_3_P)_2_PdCl_2_] (8)	dioxane	90	3	29
		CuCN (16)				
6	(*S*)-[D_1_]**13**	[(Ph_3_P)_2_PdCl_2_] (8)	toluene	90	3	53
		CuCN (16)				

**Scheme 6:**
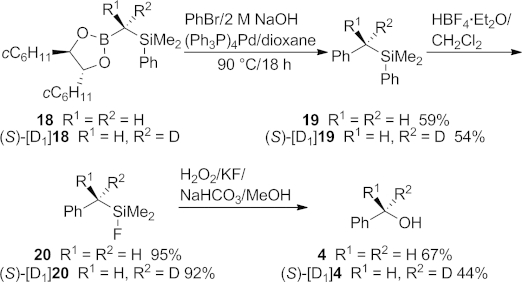
Suzuki–Miyaura reaction of boronates 18 and (*S*)-[D_1_]18.

An alternative to (tributylstannyl)[D_1_]methanol as a synthetic equivalent of chiral CHDOH is boronate (*S*)-[D_1_]**18**,[24] which is more easily accessible than the former compound (Scheme 6). To study the feasibility of the given sequence, we tested the Suzuki–Miyaura reaction[25] for transferring the [(dimethylphenyl)silyl]methyl group in non-deuterated and deuterated form from boronates **18** and (*S*)-[D_1_]**18**, respectively. The silanes would be converted into phenylmethanols **4** by the Tamao–Kumada–Fleming oxidation.[26] Surprisingly, the coupling of boronate **18** with excess bromobenzene (3 equiv.) using [(Ph_3_P)_4_Pd] (5 mol-%) as catalyst was a slow transformation. The reaction temperature had to be maintained at 90 °C and the reaction time at 18 h to obtain a yield of 59 % of silane **19**. Biphenyl was formed as an unexpected side product in 15 % yield by transferring the phenyl group from the silicon atom.[27] The best way to convert coupling product **19** into phenylmethanol (**4**), was first treatment with HBF_4_**·**Et_2_O to rapidly generate silyl fluoride **20** in high yield,[28] then oxidation[29] of the crude product to **4** in 67 % yield.

Similarly, boronate (*S*)-[D_1_]**18** furnished [D_1_]**4** in 99 % *ee* with (*S*) configuration, as determined by ^1^H NMR spectroscopic analysis of its (*R*)-Mosher ester. This result implies that the chiral [(dimethylphenyl)silyl]methyl group was transferred with net retention of configuration and that the intermediate palladium complex [{(PhMe_2_Si)CHD}PdPhL*_n_*] was configurationally stable during its (supposedly) short life-time. These results are in line with studies published by Crudden et al.,[30] who demonstrated that Pd-catalyzed cross coupling of chiral secondary benzylboronic esters followed a retentive course.

The first step in the Stille and the Miyaura–Suzuki reaction is the oxidative addition of PdL*_n_* to PhBr or PhC(O)Cl, followed by transmetalation with either a stannane or boronate to give intermediates [(XCHD)PdYL*_n_*] [X = OH, PhCO_2_, PhthN and Y = Ph or PhC(O) or X = PhMe_2_Si, Y = Ph]. As we find net retention of configuration for the coupling, transmetalation must occur via a cyclic closed transition state with retention of configuration, because reductive elimination follows a retentive course. In the case of transmetalation of [PhC(O)PdL*_n_*] with *N*-[(tributylstannyl)methyl]phthalimides **13**, either an open transition state interferes with the closed transition state, or the closed transition state is not microscopically configurationally stable, which is more pronounced in dioxane than in toluene. Accordingly, the *ee* is lowered from the usual 99 % to 52 and 69 %, respectively.

## Conclusions

We have used the Stille and, in one case, also the Suzuki–Miyaura cross-coupling to transfer chiral heteroatom-substituted [D_1_]methyl groups to bromobenzene and benzoyl chloride. [(Ph_3_P)_4_Pd] or [(Ph_3_P)_2_PdCl_2_], either alone or in combination with Cu^I^CN as cocatalyst were used as catalysts. These reactions follow a net retentive course, yielding products with 99 % *ee*; only the coupling of *N*-[(tributylstannyl)[D_1_]methyl]phthalimide with bromobenzene gave products with 52–69 % *ee*, depending on the solvent used. Therefore, except for the latter case, the intermediate chiral [XCHDPdL*_n_*] complexes must be microscopically configurationally stable (relative to reductive elimination). The results presented here, in combination with the previous studies with lithium, show nicely that the configurational stability of XCHD–metal compounds is very much dependent on the metal used.

## Experimental Section

**Stille Coupling of Bromobenzene with (Tributylstannyl)methanol (3) and (*S*)-(Tributylstannyl)[D_1_]methanol [(*S*)-[D_1_]3]:** Anhydrous 1,4-dioxane (4 mL) and bromobenzene (0.141 g, 0.9 mmol, 0.094 mL) were added to (tributylstannyl)methanol (**3**; 0.435 g, 1.35 mmol) and [Pd(PPh_3_)_4_] (0.052 g, 0.045 mmol) under argon at room temperature.[8] The mixture was stirred at 80 °C for 18 h. After cooling to room temperature, the mixture was concentrated under reduced pressure and purified by flash chromatography (hexane/EtOAc, 5:1; *R*_f_ = 0.32) to give phenylmethanol (**4**; 0.055 g, 57 %) as a colorless liquid. ^1^H NMR (400.13 MHz, CDCl_3_): *δ* = 7.36–7.32 (m, 4 H), 7.30–7.25 (m, 1 H), 4.67 (s, 2 H) ppm. ^13^C NMR (100.61 MHz, CDCl_3_): *δ* = 140.9, 128.6 (2 C), 127.7, 127.0 (2 C), 65.4 ppm. Similarly, (*S*)-(tributylstannyl)[D_1_]methanol {(*S*)-[D_1_]**3**; 0.312 g, 0.97 mmol} was converted into (*R*)-phenyl[D_1_]methanol {(*R*)-[D_1_]**4**; 0.040 g, 38 %}. ^1^H NMR (400.27 MHz, CDCl_3_): *δ* = 7.38–7.33 (m, 4 H), 7.32–7.25 (m, 1 H), 4.66 (t, *J* = 1.8 Hz, 1 H, CHD) ppm.

**Supporting Information** (see footnote on the first page of this article): General information, detailed experimental procedures and copies of the ^1^H and ^13^C NMR spectra of products.

## Notes

As a service to our authors and readers, this journal provides supporting information supplied by the authors. Such materials are peer reviewed and may be re-organized for online delivery, but are not copy-edited or typeset. Technical support issues arising from supporting information (rother than missing files) should be addressed to the authors.
