# Supplementary material for: On the Configurational Stability of Chiral Heteroatom-Substituted [D1]Methylpalladium Complexes as Intermediates of Stille and Suzuki–Miyaura Cross-Coupling Reactions
Source: European J Org Chem. 2013 Jun 27;2013(23):5143–8. doi: 10.1002/ejoc.201300439 (PMC3790956; doi:10.1002/ejoc.201300439)
Supplement: Supplementary file 1 [file ejoc2013-5143-SD1.pdf]

Eur. J. Org. Chem. **2013** · © WILEY-VCH Verlag GmbH & Co. KGaA, 69451 Weinheim, 2013 · ISSN 1099–0690

SUPPORTING INFORMATION

DOI: 10.1002/ejoc.201300439

Title: On the Configurational Stability of Chiral Heteroatom-Substituted [D_1]Methylpalladium Complexes as Intermediates of Stille and Suzuki–Miyaura Cross-Coupling Reactions

Author(s): Petra Malova Krizkova, Friedrich Hammerschmidt*

1. GENERAL **2**

2. STILLE COUPLING WITH TRIBUTYLSTANNYLMETHANOLS **2**

3. STILLE COUPLING WITH TRIBUTYLSTANNYLMETHYL BENZOATES **3**

4. ATTEMPTED STILLE COUPLING WITH (BENZYLTHIOMETHYL)TRIBUTYLSTANNANE AND (*S*)-TRIBUTYLSTANNYLMETHYL THIOACETATE **7**

5. STILLE COUPLING WITH *N*-(TRIBUTYLSTANNYLMETHYL)PHTHALIMIDES **8**

6. SUZUKI-MYIAURA COUPLING WITH (DIMETHYLPHENYLSILYLMETHYL)BORONATES **13**

7. SPECTRA **16**

8. LITERATURE **28**

1. General

Flash chromatography was performed with Merck silica gel 60 (230-400 mesh). Reactions and flash (column) chromatography were monitored by TLC carried out on 0.25 mm thick, silica gel 60, F_{254} Merck plates. The spots were visualized, unless otherwise specified, by UV and/or dipping the plate into a solution of $(NH_4)_6Mo_7O_{24}\cdot 4\ H_2O$ (23.0 g) and $Ce(SO_4)_2\cdot 4\ H_2O$ (1.0 g) in 10% aqueous H_2SO_4 (500 mL) and heating with a heat gun.

$^1H/^{13}C$ NMR spectra were measured at 300 K on Bruker Avance DRX 400 (^1H: 400.13 MHz, ^{13}C: 100.61 MHz), AV 400 (^1H: 400.27 MHz, ^{13}C: 100.65 MHz) and DRX 600 (^1H: 600.13 MHz, ^{13}C: 150. 92 MHz). The spectra were referenced to residual $CHCl_3$ (δ_H 7.24) or $CDCl_3$ (δ_C 77.00).

IR spectra were recorded on a silicon disc (Si) on a Perkin-Elmer 1600 FT-IR spectrometer or by using ATR on a Bruker VERTEX 70 IR spectrometer or Perkin-Elmer Spectrum 2000 FT-IR spectrometer.

High resolution mass spectra (HRMS) were obtained on a MAT 59 using the EI ionization method.

Optical rotations were measured at 20 °C on a Perkin-Elmer 141 polarimeter in a 1 dm cell.

Melting points were measured with a Reichert Thermovar instrument and are uncorrected.

Dry THF (over K) and Et_2O (over $LiAlH_4$) were used right after distillation. TMEDA was refluxed over CaH_2, distilled and stored over molecular sieves (4 Å).

All of the other chemicals were used as supplied from Aldrich, Fluka, Merck or Acros.

2. Stille coupling with tributylstannylmethanols

Phenyl- and (*R*)-phenyl-[D$_1$]methanol [4 and (*R*)-[D$_1$]4]

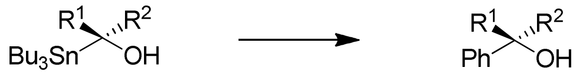

3: $R^1 = R^2 = H$ **4**: $R^1 = R^2 = H$

(*S*)-[D$_1$]**3**: R^1 = D, R^2 = H   (*R*)-[D$_1$]**4**: R^1 = D, R^2 = H

Dry 1,4-dioxane (4 mL) and bromobenzene (0.141 g, 0.9 mmol, 0.094 mL) were added to tributylstannylmethanol (**3**, 0.435 g, 1.35 mmol) and $Pd(PPh_3)_4$ (0.052 g, 0.045 mmol) under argon at RT.[1] The mixture was stirred for 18 h at 80 °C. After cooling to RT it was concentrated under reduced pressure and purified by flash chromatography (hexane:EtOAc = 5:1, R_f = 0.32) to yield phenylmethanol

(**4**, 0.055 g, 57%) as a colorless liquid. ^{1}H NMR (400.13 MHz, $CDCl_3$): δ 7.36-7.32 (m, 4H), 7.30-7.25 (m, 1H), 4.67 (s, 2H); ^{13}C (100.61 MHz, $CDCl_3$): δ 140.9, 128.6 (2C), 127.7, 127.0 (2C), 65.4.

Similarly, (*S*)-tributylstannyl-[D_1]methanol [(*S*)-[D_1]**3**, 0.312 g, 0.97 mmol] was converted to (*R*)-phenyl-[D_1]methanol [(*R*)-[D_1]**4**, 0.040 g, 38%]. ^{1}H NMR (400.27 MHz, $CDCl_3$): δ 7.38-7.33 (m, 4H), 7.32-7.25 (m, 1H), 4.66 (t, J = 1.8 Hz, 1H, CHD).

(*R*)-Mosher ester of (*R*)-phenyl-[D_1]methanol [(*R*)-[D_1]4·MTPA-(*R*)]

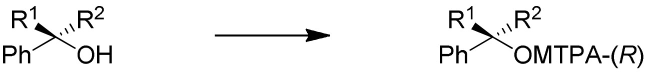

(*R*)-[D_1]**4**: R^1 = D, R^2 = H (*R*)-[D_1]**4**·MTPA-(*R*): R^1 = D, R^2 = H

A mixture of dry pyridine (0.480 g, 6.1 mmol, 0.49 mL), (*S*)-MTPACl (0.295 mmol, 0.60 mL, 0.53M in CH_2Cl_2) and (*R*)-phenyl-[D_1]methanol [(*R*)-[D_1]**4**, 0.021 g, 0.19 mmol] in dry CH_2Cl_2 (4 mL) was stirred for 18 h under argon at RT. After the addition of water (5 mL) stirring was continued for another 5 min. CH_2Cl_2 and HCl (1M) were added and the phases were separated. The aqueous layer was extracted twice with CH_2Cl_2. The combined organic layers were washed with a saturated aqueous solution of $NaHCO_3$, dried (Na_2SO_4) and concentrated under reduced pressure. The crude product was purified by flash chromatography (hexane:EtOAc = 10:1, R_f = 0.38) to yield Mosher ester of (*R*)-[D_1]**4** (0.033 g, 53%, ee $\geq$ 99%) as a colorless oil; ^{1}H NMR (400.27 MHz, $CDCl_3$): δ 7.46-7.41 (m, 2H), 7.40-7.30 (m, 8H), 5.33 (broadened s, 1H, CHD), 3.50 (q, J = 1.3 Hz, 3H).

Similarly, (*S*)-phenyl-[D_1]methanol obtained by horse-liver alcohol dehydrogenase catalyzed reduction of [formyl-D_1]benzaldehyde was converted to its (*R*)-Mosher ester. ^{1}H NMR (400.27 MHz, $CDCl_3$): δ 7.45-7.31 (m, 10H), 5.29 (t, J_{HD} = 1.5 Hz, 1H, CHD), 3.50 (q, J_{HF} = 1.1 Hz, 3H).

3. Stille coupling with tributylstannylmethyl benzoates

Tributylstannylmethyl-, (*R*)- and (*S*)-tributylstannyl-[D_1]methyl benzoate [5, (*R*)- and (*S*)-[D_1]5]

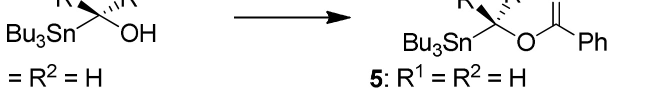

3: R^1 = R^2 = H
(*R*)-[D_1]**3**: R^1 = H, R^2 = D
(*S*)-[D_1]**3**: R^1 = D, R^2 = H

5: R^1 = R^2 = H
(*R*)-[D_1]**5**: R^1 = H, R^2 = D
(*S*)-[D_1]**5**: R^1 = D, R^2 = H

A mixture of dry CH_2Cl_2 (4.6 mL), dry pyridine (0.487 g, 6.16 mmol, 0.496 mL), benzoyl chloride (0.433 g, 2.08 mmol, 0.358 mL) and tributylstannylmethanol (**3**, 0.496 g, 1.54 mmol) was stirred for 20 h under argon at RT. After the addition of water (2 mL) it was stirred for another 10 min. HCl (1M) and CH_2Cl_2 were added and the phases were separated. The aqueous layer was extracted with CH_2Cl_2 and the combined organic layers were washed with a saturated solution of $NaHCO_3$, dried ($MgSO_4$) and concentrated under reduced pressure. The crude product was purified by flash chromatography (hexane:CH_2Cl_2 = 1:1, R_f = 0.80) to yield benzoate **5** (0.551g, 84%) as a colorless oil. IR (ATR): ν 2955, 2922, 1708, 1315, 1290, 1105; ^{1}H NMR (400.27 MHz, $CDCl_3$): δ 8.01-7.95 (m, 2H), 7.54-7.49 (m, 1H), 7.43-7.37 (m, 2H), 4.41 (s, $J(^{117/119}Sn)$ = 11.8 Hz, 2H), 1.61-1.41 (m, 6H), 1.27 (sext, J = 7.3 Hz, 6H), 1.03-0.86 (m, 6H), 0.85 (t, J = 7.3 Hz, 9H); ^{13}C NMR (100.61 MHz, $CDCl_3$): δ 132.5, 129.4 (2C), 128.2 (2C), 56.4, 29.0 ($J(^{117/119}Sn)$ = 21.2 Hz, 3C), 27.3 ($J(^{117/119}Sn)$ = 53.4 Hz, 3C), 13.7, 9.7 ($J(^{117/119}Sn)$ = 319.3 Hz, 3C), C=O and C_q n. d.

Similarly, (*R*)-tributylstannyl-[D_1]methanol [(*R*)-[D_1]**3**, 0.327 g, 1.01 mmol] was converted to (*R*)-tributylstannyl-[D_1]methyl benzoate [(*R*)-[D_1]**5**, 0.369 g, 90%, $[\alpha]_D^{20}$ = -1.24 (c = 18.5, acetone).

Similarly, (*S*)-tributylstannyl-[D_1]methanol [(*S*)-[D_1]**3**, 0.334 g, 1.00 mmol] was converted to (*S*)-tributylstannyl-[D_1]methyl benzoate [(*S*)-[D_1]**5**, 0.351 g, 82%].

^{1}H and ^{13}C NMR spectra for (*R*)- and (*S*)-1-tributylstannyl-[D_1]methanyl benzoate were identical: ^{1}H NMR (400.27 MHz, $CDCl_3$): δ 8.01-7.95 (m, 2H), 7.54-7.48 (m, 1H), 7.43-7.38 (m, 2H), 4.39 (broadened s, 1H, CHD),1.61-1.41 (m, 6H), 1.27 (sext, J = 7.3 Hz, 6H), 1.03-0.86 (m, 6H), 0.85 (t, J = 7.3 Hz, 9H); ^{13}C NMR (100.61 MHz, $CDCl_3$): δ 132.5, 129.3 (2C), 128.3 (2C), 56.1 (t, J = 22.3 Hz, CHD), 29.0 ($J(^{117/119}Sn)$ = 21.2 Hz, 3C), 27.3 ($J(^{117/119}Sn)$ = 53.4 Hz, 3C), 13.7, 9.7 ($J(^{117/119}Sn)$ = 319.3 Hz, 3C), C=O and C_q n. d.

2-Phenyl-2-oxoethyl-, (*R*)- and (*S*)-2-phenyl-2-oxo-[1-D_1]ethyl benzoate [6, (*R*)- and (*S*)-[1-D_1]6]

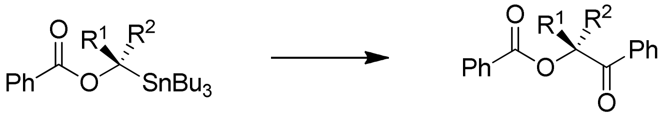

5: $R^1 = R^2$ = H
(*R*)-[D_1]**5**: R^1 = D, R^2 = H
(*S*)-[D_1]**5**: R^1 = H, R^2 = D

6: $R^1 = R^2$ = H
(*S*)-[1-D_1]**6**: R^1 = D, R^2 = H
(*R*)-[1-D_1]**6**: R^1 = H, R^2 = D

A mixture of benzoyl chloride (0.116 g, 0.82 mmol, 0.095 mL) tributylstannylmethyl benzoate (**5**, 0.366 g, 0.86 mmol), $(PPh_3)_2PdCl_2$ (25.3 mg, 0. 039 mmol, 4.5 mol%) and CuCN (8.1 mg, 0.09 mmol, 10.5 mol%) in dry toluene (8 mL) was stirred under argon at 70 °C for 18 h.[2] After cooling to RT it was concentrated under reduced pressure and purified by flash chromatography (hexane:EtOAc= 5:1, R_f = 0.53) to yield benzoate **6** (0.138 g, 67%, contained 28 mol% of an impurity possibly formed from the staring material losing a butyl group) as a crystalline solid. IR (Si): ν 2956, 2926, 1720, 1701, 1284; ^{1}H NMR (400.13 MHz, $CDCl_3$): δ 8.15-8.11 (m, 2H), 8.09-8.03 (m, 1H), 7.97-7.93 (m, 1H), 7.65-7.55 (m, 2H), 7.52-7.43 (m, 4H), 5.56 (s, 2H), significant signals of impurity: 4.14 (s, $J(^{117/119}Sn)$ = 27.5 Hz, 2H), and signals for two butyl groups on Sn.

Similarly, (*S*)-tributylstannyl-[D_1]methyl benzoate [(*S*)-[D_1]**5**, 0.188 g, 0.44 mmol] was converted to (*R*)-2-phenyl-2-oxo-[1-D_1]ethyl benzoate [(*R*)-[1-D_1]**6**, 0.100 g, 94%, contained 18 mol% of Bu_3SnX and 2 mol% of the unlabeled species].

Similarly, (*R*)-tributylstannyl-[D_1]methyl benzoate [(*R*)-[D_1]**5**, 0.188 g, 0.44 mmol] was converted to (*S*)-2-phenyl-2-oxo-[1-D_1]ethyl benzoate [(*S*)-[1-D_1]**6**, 0.100 g, 94%, contained 26 mol% of the same impurity as the unlabeled species and 2 mol% of the unlabeled species].

^{1}H NMR spectra for (*R*)- and (*S*)-2-phenyl-2-oxo-[1-D_1]ethyl benzoate were identical: ^{1}H NMR (400.27 MHz, $CDCl_3$): δ 8.16-8.10 (m, 2H), 7.98-7.93 (m, 2H), 7.62-7.55 (m, 2 H), 7.52-7.42 (m, 4H), 5.56 (s of the unlabeled species), 5.54 (t, J = 2.3 Hz, 1H, CHD).

1-Phenyl-, (1*RS*,2*R*)- and (1*RS*,2*S*)-1-phenyl-1,2-[2-D_1]ethanediol [7, (1*RS*,2*R*)- and (1*RS*, 2*S*)-[2-D_1]7]

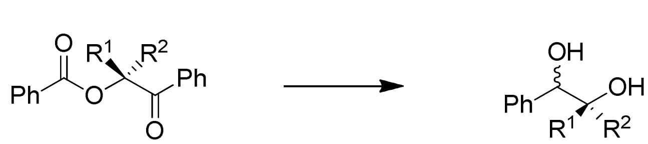

6: $R^1 = R^2$ = H
(*R*)-[1-D_1]**6**: R^1 = H, R^2 = D
(*S*)-[1-D_1]**6**: R^1 = D, R^2 = H

7: $R^1 = R^2$ = H
(2*R*)-[2-D_1]**7**: R^1 = H, R^2 = D
(2*S*)-[2-D_1]**7**: R^1 = D, R^2 = H

A solution of 2-phenyl-2-oxoethyl benzoate (**6**, 0.132 g, 0.50 mmol) in dry THF (2 mL) was added drop wise to $LiAlH_4$ (2 mL, 2M in THF) under argon at 0 °C. It was then stirred at RT for 1 h. After cooling to 0 °C, EtOAc (2 mL) was carefully added followed by more EtOAc and HCl (2M) were added after 15 min at 0 °C and the phases were separated. The aqueous layer was extracted twice with EtOAc. The

combined organic layers were washed with a saturated aqueous solution of $NaHCO_3$, dried (Na_2SO_4) and concentrated under reduced pressure. The residue was purified by flash chromatography (hexane:EtOAc = 1:1, R_f = 0.24) to yield crystalline diol **7** (0.022 g, 32%). ^{1}H NMR (400.13 MHz, $CDCl_3$): δ 7.37-7.34 (m, 8H), 7.31-7.28 (m, 2H), 4.82 (d, J = 4.7 Hz, 1H), 4.82 (d, J = 11.5 Hz, 1H), 3.76 (dd, J = 11.6, 4.0 Hz, 2H), 3.66 (dd, J = 11.2, 8.2 Hz, 2H).

Similarly, (*R*)-2-phenyl-2-oxo-[1-D_1]ethyl benzoate [(*R*)-[1-D_1]**6**, 0.095 g, 0.39 mmol] was converted to (*R*)-1-phenyl-1,2-[2-D_1]ethanediol [(1*RS*,2*R*)-[2-D_1]**7**, 0.032 g, 59%].

Similarly, (*S*)-2-phenyl-2-oxo-[1-D_1]ethyl benzoate [(*S*)-[1-D_1]**6**, 0.104 g, 0.43 mmol] was converted to (*S*)-1-phenyl-1,2-[2-D_1]ethanediol [(1*RS*,2*S*)-[2-D_1]**7**, 0.02 g, 37%].

^{1}H and ^{13}C NMR spectra for (*R*)- and (*S*)-1-phenyl-1,2-[2-D_1]ethanediol were identical: ^{1}H NMR (400.27 MHz, $CDCl_3$): δ 7.37-7.34 (m, 8H), 7.31-7.28 (m, 2H), 4.80 (d, J = 8.0 Hz, 1H), 4.80 (d, J = 3.7 Hz, 1H), 3.73 (td, J = 1.6 Hz, 3.3 Hz, 1H, CHD), 3.63 (td, J = 1.5 Hz, 8.2 Hz, 1H, CHD); ^{13}C NMR (100.65 MHz, $CDCl_3$): δ 140.5, 128.6 (2C), 128.0, 126.0 (2C), 74.62 and 74.61 (CH of the two diastereomers), 67.75 (t, J = 20.8 Hz) and 67.69 (t, J = 22.7 Hz) (CHD of the two diastereomers).

(*R*)-Mosher esters of (1*RS*,2*R*)- and (1*RS*,2*S*)-1-phenyl-1,2-[2-D_1]ethanediol [(1*RS*,2*R*)- and (1*RS*, 2*S*)-[2-D_1]7·MTPA-(*R*)]

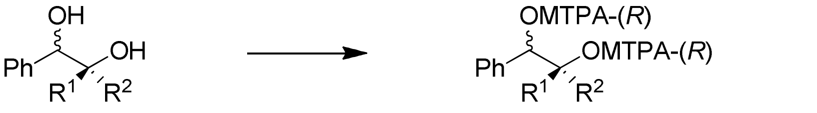

(2*R*)-[2-D_1]**7**: R^1 = H, R^2 = D
(2*S*)-[2-D_1]**7**: R^1 = D, R^2 = H

(2*R*)-[2-D_1]**7**·MTPA-(*R*): R^1 = H, R^2 = D
(2*S*)-[2-D_1]**7**·MTPA-(*R*): R^1 = D, R^2 = H

A mixture of dry pyridine (0.40 mL), (*S*)-MTPACl (1.14 mL of a 0.53M solution in dry CH_2Cl_2) and (1*RS*,2*R*)-1-phenyl-1,2-[2-D_1]ethanediol [(1*RS*,2*R*)-[2-D_1]**7**, 0.019 g, 0.14 mmol] in dry CH_2Cl_2 (3 mL) was stirred under argon at RT for 18 h. After the addition of water (0.40 mL) and stirring for 5 min, CH_2Cl_2 and HCl (2M) were added. The phases were separated and the aqueous layer was extracted twice with CH_2Cl_2. The combined organic layers were washed with a saturated aqueous solution of $NaHCO_3$, dried (Na_2SO_4) and concentrated under reduced pressure. The residue was purified by flash chromatography (hexane:EtOAc = 10:1, R_f = 0.38) to yield a 1:1 mixture of diastereomeric (*R*)-Mosher esters (1*RS*,2*R*)-[2-D_1]**7**·MTPA-(*R*) (0.049 g, 61%, ee > 99%, contained 2% of the unlabeled species) as a colorless oil. ^{1}H NMR (400.27 MHz, $CDCl_3$): δ 7.45-7.41 (m, 2H), 7.39-7.26 (m, 26H), 7.20-7.16 (m,

2H), 6.26 (d, J = 3.9 Hz, 1H), 6.14 (d, J = 8.7 Hz, 1H), 4.63 (broadened d, J = 3.9 Hz, 1H, CHD), 4.47 (broadened d, J = 8.7 Hz, 1H, CHD), 3.45 (q, J = 1.0 Hz, 3H), 3.38 (q, J = 1.0 Hz, 3H), 3.34 (q, J = 1.0 Hz, 3H), 3.32 (q, J = 1.3 Hz, 3H).

Similarly, (1*RS*,2*S*)-1-phenyl-1,2-[2-D_1]ethanediol [(1*RS*,2*S*)-[2-D_1]**7**, 0.010 g, 0.07 mmol] was converted to a 1:1 mixture of (*R*)-Mosher esters of the (1*RS*,2*S*)-1-phenyl-1,2-[2-D_1]ethanediol [(1*RS*,2*S*)-[2-D_1]**7**·MTPA-(*R*), 0.028 g, 70%, ee > 99%, contained 2% of the unlabeled species]. ^{1}H NMR (400.27 MHz, $CDCl_3$): δ 7.45-7.41 (m, 2H), 7.39-7.26 (m, 26H), 7.20-7.16 (m, 2H), 6.26 (d, J = 7.4 Hz, 1H), 6.15 (d, J = 2.8 Hz, 1H), 4.64 (broadened d, J = 2.8 Hz, 1H, CHD), 4.46 (broadened d, J = 7.4 Hz, 1H, CHD), 3.45 (q, J = 1.1 Hz, 3H), 3.39 (q, J = 1.1 Hz, 3H), 3.35 (q, J = 1.0 Hz, 3H), 3.34 (q, J = 1.1 Hz, 3H).

4. Attempted Stille coupling with (benzylthiomethyl)tributylstannane and (*S*)-tributylstannylmethyl thioacetate

(Benzylthiomethyl)tributylstannane (10)

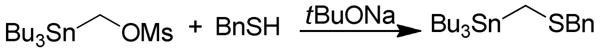

8 **9** **10**

Benzylmercaptane (**9**, 0.35 mL, 3.0 mmol) was added to a mixture of *t*-BuONa (289 mg, 3.0 mmol) in dry THF (6.3 mL) and stirred under argon for 10 min. [3] Afterwards the reaction mixture was cooled to –30 °C and tributylstannyl mesylate[4] (798 mg, 2.0 mmol) in dry THF (3.5 mL) was added. Stirring was continued for 15 min, then 1M HCl (10 mL) and hexane (10 mL) were added. The organic phase was separated and washed with brine (20 mL), dried ($MgSO_4$) and concentrated under reduced pressure. The residue was purified first by bulb-to-bulb distillation (45 °C, 0.5 mbar) to remove excess benzylmercaptane and then by flash chromatography (hexane/CH_2Cl_2 = 30:1, R_f= 0.33) to yield stannane **10** (786 mg, 92%) as a colorless oil. ^{1}H NMR (400.27 MHz, $CDCl_3$): δ 7.35-7.15 (m, 5H), 3.64 (s, 2H), 1.77 (s, $J(^{117/119}Sn)$ = 41.6 Hz, 2H), 1.57-1.33 (m, 6H), 1.30 (sext, J = 7.3 Hz, 6H), 0.97-0.80 (m, $J(^{117/119}Sn)$ = 49.6 Hz, 6H), 0.85 (t, J = 7.3 Hz, 9H).

S-Tributylstannylmethyl thioacetate (11)

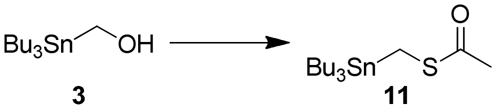

3 **11**

A mixture of triphenylphosphane (0.882 g, 3.36 mmol) and DIAD (0.680 g, 3.36 mmol, 0.662 mL) in dry THF (8 mL) was stirred for 30 min under argon before a solution of tributylstannylmethanol (**3**, 0.864 g, 2.69 mmol) and thioacetic acid (0.409 g, 5.28 mmol, 0.385 mL) in dry THF (3 mL) was added.[5] The solution color changed from yellow to green and then turned back to yellowish brown after a few minutes. Stirring was continued for 16 h and then the reaction mixture was concentrated under reduced pressure. The residue was purified by flash chromatography (hexane:CH_2Cl_2 = 10:1, R_f = 0.11) to give the thioacetate **11** (0.852 g, 84%) as a colorless oil. IR (ATR): 2956, 2921, 1738, 1692, 1675, 1457, 1376, 1217, 1131; ^{1}H NMR (400. 27 MHz, $CDCl_3$): δ 2.29 (s, 3H), 2.04 (s, $J(^{117/119}Sn)$ = 34.4 Hz, 2H), 1.59-1.38 (m, 6H), 1.29 (sext, J = 7.3 Hz, 6H), 1.00-0.82 (m, 6H), 0.88 (t, J = 7.3 Hz, 9H); ^{13}C NMR (100.65 MHz, $CDCl_3$): δ 199.0, 29.8, 28.9 (J($^{117/119}$Sn) = 28.0 Hz, 3C), 27.3 ($J(^{117/119}Sn)$ = 56.1 Hz, 3C), 13.7 (3C), 10.0 ($J(^{117/119}Sn)$ = 341.6, 321.2 Hz, 3C), 4.99 ($J(^{117/119}Sn)$ = 216.7 Hz); anal. calcd. for $C_{15}H_{32}OSSn$: C 47.51%, H 8.51%, S 8.46%; found: C 47.48%, H 8.44%, S 8.57%.

5. Stille coupling with *N*-(tributylstannylmethyl)phthalimides

1-Phenyl-2-phthalimido- and (*S*)-1-phenyl-2-phthalimido-[2-D$_1$]ethan-1-one [14 and (*S*)-[2-D$_1$]14]

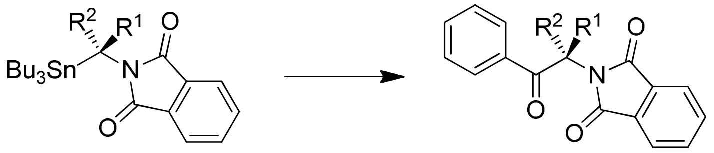

13: R^1 = R^2 = H
(*R*)-[D$_1$]**13**: R^1 = H, R^2 = D

14: R^1 = R^2 = H
(*S*)-[D$_1$]**14**: R^1 = H, R^2 = D

A mixture of benzoyl chloride (0.067 g, 0.475 mmol, 0.054 mL), *N*-(tributylstannylmethyl)phthalimide[5] (**13**, 0.336 g, 0.5 mmol), $(PPh_3)_4Pd$ (23.1 mg, 0.02 mmol, 4 mol%) and CuCN (4.7 mg, 0.04 mmol, 8 mol%) in dry toluene (6 mL) was stirred under argon at 75 °C for 20 h.[2] After cooling it was concentrated under reduced pressure and the residue was purified by flash chromatography (CH_2Cl_2, R_f = 0.31) to yield the substituted phthalimide[6] **14** (0.104 g, 78%) as colorless crystalline solid. ^{1}H NMR (400.27 MHz, $CDCl_3$): δ 8.04-7.97 (m, 2H), 7.91-7.86 (m, 2H), 7.77-7.71 (m, 2H), 7.64-7.60 (m, 1H), 7.53-7.48 (m, 2H), 5.11 (s, 2H); ^{13}C NMR (100.65 MHz, $CDCl_3$): δ 191.0, 167.9 (2C), 134.5, 134.1 (2C), 134.0, 132.3 (2C), 128.9 (2C), 128.2 (2C), 123.6 (2C), 44.2.

Similarly, (*R*)-*N*-(tributylstannyl-[D$_1$]methyl)phthalimide[5] [(*R*)-[D$_1$]**13**, 0.242 g, 0.53 mmol] was converted to (*S*)-1-phenyl-2-phthalimido-[2-D$_1$]ethan-1-one [(*S*)-[2-D$_1$]**14**, 0.109 g, 77%]. ^{1}H NMR (400.27 MHz, $CDCl_3$): δ 8.02-7.97 (m, 2H), 7.90-7.86 (m, 2H), 7.79-7.71 (m, 2H), 7.64-7.59 (m, 1H),

7.55-7.48 (m, 2H), 5.10 (t, J = 2.1 Hz, 1H, CHD); ^{13}C NMR (100.65 MHz, $CDCl_3$): δ 191.0, 167.9 (2C), 134.5, 134.1 (2C), 134.0, 132.3 (2C), 128.9 (2C), 128.2 (2C), 123.6 (2C), 50.0 (t, J = 21.4 Hz, NCHD).

2-Phthalimido-1-phenyl- and (1*RS*,2*S*)-2-phthalimido-1-phenyl-[2-D$_1$]ethanol [15 and (1*RS*,2*S*)-[2-D$_1$]15]

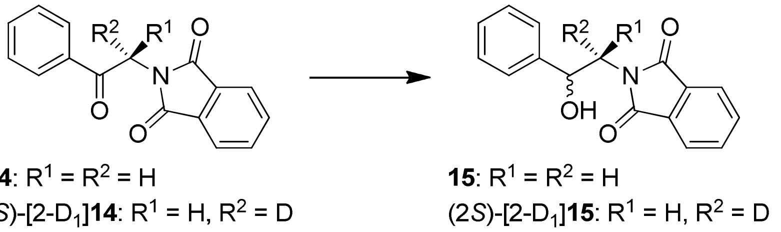

A mixture of 1-phenyl-2-phthalimidoethan-1-one (**14**, 0.133 g, 0.5 mmol) and 10% Pd/C (66.3 mg, 50 weight %) in dry EtOAc (p. A., 15 mL) was hydrogenated in a Parr apparatus (4 h, 60 psi, RT).[7] The mixture was filtered and concentrated under reduced pressure. The residue was recrystallized from CH_2Cl_2/$i$$Pr_2O$ to give the alcohol **15** (0.103 g, 0.39 mmol, 77%) as colorless crystals; mp. 166-167 °C (lit.: [8] 164-165°C). ^{1}H NMR (400.27 MHz, $CDCl_3$): δ 7.87-7.81 (m, 2H), 7.73-7.68 (m, 2H), 7.47-7.40 (m, 2H), 7.37-7.32 (m, 2H), 7.30-7.25 (m, 1H), 5.06 (dd, J = 8.4, 3.7 Hz, 1H), 3.97 (AB part of the ABX-system, J_{AB} = 14.2 Hz, J_{AX} = 8.4 Hz, J_{BX} = 3.7 Hz, 2H), 2.80 (br. s, 1H); ^{13}C NMR (100.65 MHz, $CDCl_3$): δ 168.8 (2C), 141.1, 134.1 (2C), 131.9 (2C), 128.6 (2C), 128.1, 125.9 (2C), 123.5 (2C), 72.7, 45.8.

Similarly, (*S*)-1-phenyl-2-phthalimido-[2-D$_1$]ethan-1-one [(*S*)-[2-D$_1$]**14**, 0.052 g, 0.20 mmol] was converted to (1*RS*,2*S*)-2-phthalimido-1-phenyl-[2-D$_1$]ethanol [(1*RS*,2*S*)-[2-D$_1$]**15**, 0.021 g, 41%). ^{1}H NMR (400.27 MHz, $CDCl_3$): δ 7.87-7.81 (m, 4H), 7.73-7.68 (m, 4H), 7.47-7.40 (m, 4H), 7.37-7.32 (m, 4H), 7.30-7.25 (m, 2H), 5.05 (d, J = 3.7 Hz, 1H), 5.05 (d, J = 8.3 Hz, 1H), 3.99 (d, J = 8.8 Hz, 1H, CHD), 3.92 (broadened s, 1H, CHD); ^{13}C NMR (100.65 MHz, $CDCl_3$): δ 168.7 (2C), 141.1, 134.1 (2C), 131.9 (2C), 128.6 (2C), 128.1, 125.9 (2C), 123.5 (2C), 72.7, 45.5 (t, J = 21.6 Hz, NCHD).

(*R*)-Mosher esters of 2-phthalimido-1-phenyl- and (1*RS*,2*S*)-2-phthalimido-1-phenyl-[2-D_1]ethanol [15a and (1*RS*,2*S*)-[2-D_1]15·MTPA-(*R*)]

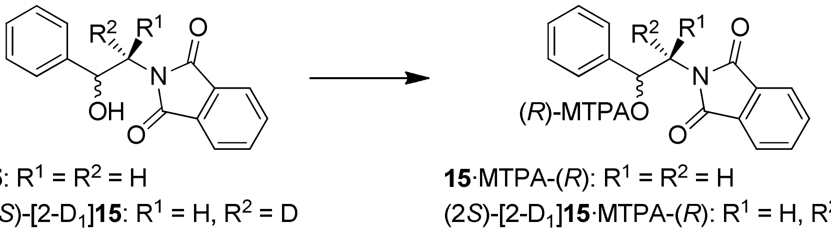

15: $R^1 = R^2 =$ H
(2*S*)-[2-D_1]**15**: R^1 = H, R^2 = D

15·MTPA-(*R*): $R^1 = R^2 =$ H
(2*S*)-[2-D_1]**15**·MTPA-(*R*): R^1 = H, R^2 = D

A solution of (*S*)-MTPACl (0.09 mmol, 0.171 mL of a solution 0.53M in dry CH_2Cl_2), 2-phthalimido-1-phenylethanol (**15**, 11.9 mg, 0.045 mmol), dry CH_2Cl_2 (0.5 mL) and pyridine (3 drops) was stirred under argon at RT for 18 h. It was concentrated under reduced pressure and water was added. The mixture was extracted three times with CH_2Cl_2 and the combined organic layers were washed with HCl (2M), a saturated aqueous solution of $NaHCO_3$, dried (Na_2SO_4) and concentrated under reduced pressure. The crude product was purified by flash chromatography (hexane:EtOAc = 5:1, R_f = 0.32) to give a 1:1 diastereomeric mixture of (*R*)-Mosher esters **15**·MTPA-(*R*) (19.8 mg, 91%) as colorless oil. ^{1}H NMR (400.27 MHz, $CDCl_3$): δ 7.85-7.80 (m, 2H), 7.77-7.68 (m, 5H), 7.51-7.46 (m, 2H), 7.42-7.30 (m, 8H), 7.28-7.20 (m, 6H), 7.15-7.08 (m, 3H), 7.07-7.00 (m, 2H), 6.34 (dd, *J* = 10.2, 4.1 Hz, 1H), 6.28 (dd, *J* = 9.8, 4.3 Hz, 1H), 4.27 (dd, *J* = 14.4, 9.8 Hz, 1H), 4.26 (dd, *J* = 14.4, 10.2 Hz, 1H), 3.86 (dd, *J* = 14.4, 4.2 Hz, 1H), 3.79 (dd, *J* = 14.4, 4.2 Hz, 1H), 3.39 (q, *J* = 1.1 Hz, 3H), 3.36 (q, *J* = 1.1 Hz, 3H).

Similarly, (1*RS*,2*S*)-2-phthalimido-1-phenyl-[2-D_1]ethanol [(1*RS*,2*S*)-[2-D_1]**15**, 0.010 g, 0.038 mmol] was converted to the (*R*)-Mosher esters (1*RS*,2*S*)-[2-D_1]**15**·MTPA-(*R*) (0.013 g, 71%, de > 98%). ^{1}H NMR (600.13 MHz, $CDCl_3$): δ 7.85-7.80 (m, 2H), 7.77-7.68 (m, 5H), 7.51-7.46 (m, 2H), 7.42-7.30 (m, 8H), 7.28-7.20 (m, 6H), 7.15-7.08 (m, 3H), 7.07-7.00 (m, 2H), 6.33 (d, *J* = 10.2 Hz, 1H), 6.28 (d, *J* = 4.2 Hz, 1H), 4.24 (d, *J* = 10.2 Hz, 1H), 3.79 (d, *J* = 4.2 Hz, 1H), 3.39 (q, *J* ~ 1 Hz, 3H), 3.36 (q, *J* ~ 1 Hz, 3H).

***N*-Phenyl-, (*R*)- and (*S*)-*N*-(phenyl-[D_1]methyl)phthalimide [16, (*R*)- and (*S*)-[D_1]16]**

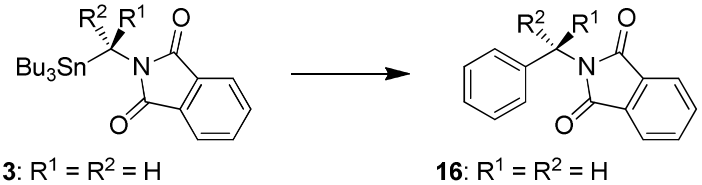

13: $R^1 = R^2 =$ H
(*R*)-[D_1]**13**: R^1 = H, R^2 = D
(*S*)-[D_1]**13**: R^1 = D, R^2 = H

16: $R^1 = R^2 =$ H
(*S*)-[D_1]**16**: R^1 = H, R^2 = D
(*R*)-[D_1]**16**: R^1 = D, R^2 = H

A mixture of bromobenzene (0.117 g, 0.75 mmol, 0.079 mL), *N*-(tributylstannylmethyl)phthalimide (**13**, 0.225 g, 0.5 mmol), $Pd(PPh_3)_2Cl_2$ (28 mg, 0.04 mmol, 8 mol%) and CuCN (7.1 mg, 0.08 mmol, 16 mol%) in dry dioxane (6 mL) was stirred under argon at 10 °C for 6 h.[2] After cooling it was concentrated under reduced pressure and a 5% aqueous solution of KF was added. The mixture was extracted twice with CH_2Cl_2. The combined organic layers were dried (Na_2SO_4) and concentrated under reduced pressure. The crude product was purified by flash chromatography (CH_2Cl_2, R_f = 0.44) to yield the *N*-(phenylmethyl)phthalimide[9] [**16**, 0.043 g, 37%] as colorless crystals. ^{1}H NMR (400.27 MHz, $CDCl_3$): δ 7.86-7.80 (m, 2H), 7.70-7.65 (m, 2H), 7.42-7.38 (m, 2H), 7.32-7.20 (m, 3H), 4.83 (s, 2H); ^{13}C NMR (100.65 MHz, $CDCl_3$): δ 168.0 (2C), 136.4, 133.9 (2C), 132.2 (2C), 128.7 (2C), 128.6 (2C), 127.8, 123.3 (2C), 41.6.

Similarly, (*S*)-*N*-(tributylstannyl-[D$_1$]methyl)phthalimide [(*S*)-[D$_1$]**13**, 0.301 g, 0.67 mmol] was converted in dry toluene to (*R*)-*N*-(phenyl-[D$_1$]methyl)phthalimide [(*R*)-[D$_1$]**16**, 0.084 g, 53%].

Similarly, (*R*)-*N*-(tributylstannyl-[D$_1$]methyl)phthalimide [(*R*)-[D$_1$]**13**, 0.206 g, 0.46 mmol] was converted in dry dioxane to (*S*)-*N*-(phenyl-[D$_1$]methyl)phthalimide [(*S*)-[D$_1$]**16**, 0.031 g, 29%].

The ^{1}H NMR spectra of (*R*)- and (*S*)-*N*-(phenyl-[D$_1$]methyl)phthalimide were identical: ^{1}H NMR (400.27 MHz, $CDCl_3$): δ 7.86-7.80 (m, 2H), 7.71-7.66 (m, 2H), 7.44-7.39 (m, 2H), 7.32-7.22 (m, 3H), 4.83-4.80 (broadened s, 1H, CHD).

(*R*)-*N*-(Phenyl-[D$_1$]methyl)phthalimide prepared from (*S*)-phenyl-[D$_1$]methanol

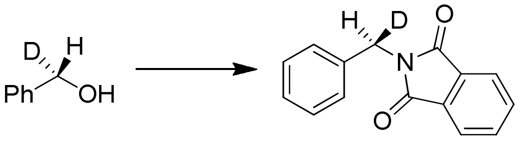

(*S*)-[D$_1$]**4** (*R*)-[D$_1$]**16**

DIAD (1.03 mmol, 0.203 mL) was added to a stirred mixture of (*R*)-phenyl-[D$_1$]methanol [(*R*)-[D$_1$]**4**, 0.094 g, 0.86 mmol], triphenylphosphane (0.270 g, 10.03 mmol) and phthalimide (0.150 g, 1.03 mmol) in dry THF (8 mL) under argon at 0 °C and stirring was continued for 2 days at RT.[5] After water (0.5 mL) was added it was concentrated under reduced pressure. The crude product was purified by flash chromatography (hexane:EtOAc = 2:1, R_f = 0.73) to give the substituted phthalimide [(*S*)-[D$_1$]**16**, 0.148 g, 72%] as colorless crystals. The NMR spectra were identical to those of the labeled products obtained by Stille coupling.

Phenyl-, (*R*)- and (*S*)-phenyl-[D_1]methylamine [17, (*R*)- and (*S*)-[D_1]17]

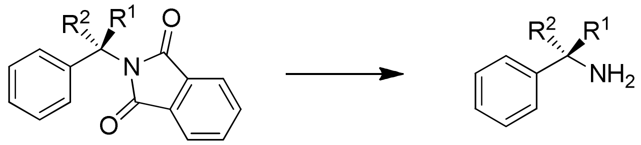

16: $R^1 = R^2 = H$
(*R*)-[D_1]**16**: $R^1 = D$, $R^2 = H$
(*S*)-[D_1]**16**: $R^1 = H$, $R^2 = D$

17: $R^1 = R^2 = H$
(*R*)-[D_1]**17**: $R^1 = D$, $R^2 = H$
(*S*)-[D_1]**17**: $R^1 = H$, $R^2 = D$

A mixture of hydrazine hydrate (0.327 g, 5.03 mmol, 0.306 mL) and *N*-(phenylmethyl)phthalimide (**16**, 0.194 g, 0.8 mmol) in dry ethanol (7 mL) was refluxed for 2 h. After cooling it was centrifuged to remove the crystalline byproduct. The pellet was washed with Et_2O and the combined organic layers were concentrated under reduced pressure. Et_2O (10 mL) and NH_3 (25%, 3 drops) were added and the mixture was first washed with a half saturated aqueous solution of NaCl (2 mL) and then with a saturated aqueous solution of NaCl (2 mL).[10] After the addition of dry ethanol (5 mL) to the organic layer it was concentrated under reduced pressure. Then dry CH_2Cl_2 (5 mL) was added, the mixture was filtered and the filtrate was concentrated under reduced pressure and dried in vacuo (0.5 mbar, 3 min) to give the amine **17** (18 mg, 21%) as a colorless liquid which was immediately derivatized with (*S*)-MTPACl in case of labeled species.

Similarly, (*R*)-*N*-(phenyl-[D_1]methyl)phthalimide [(*R*)-[D_1]**16**, 0.148 g, 0.62 mmol] was converted to (*R*)-phenyl-[D_1]methylamine [(*R*)-[D_1]**17**, 0.037 g, 55%].

Similarly, (*S*)-*N*-(phenyl-[D_1]methyl)phthalimide [(*S*)-[D_1]**16**, 0.030 g, 0.12 mmol] was converted to (*S*)-phenyl-[D_1]methylamine [(*S*)-[D_1]**17**, 0.014 g, 99%].

(*R*)-Mosher amides of phenyl-, (*R*)- and (*S*)-phenyl-[D_1]methylamine [17a, (*R*)- and (*S*)-[D_1]17·MTPA-(*R*)]

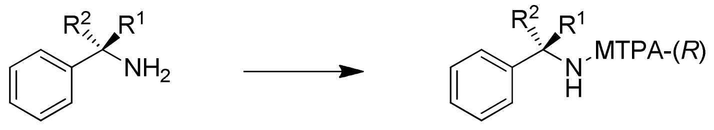

17: $R^1 = R^2 = H$
(*R*)-[D_1]**17**: $R^1 = D$, $R^2 = H$
(*S*)-[D_1]**17**: $R^1 = H$, $R^2 = D$

17a: $R^1 = R^2 = H$
(*R*)-[D_1]**17**·MTPA-(*R*): $R^1 = D$, $R^2 = H$
(*S*)-[D_1]**17**·MTPA-(*R*): $R^1 = H$, $R^2 = D$

A mixture of phenylmethylamine (**17**, 12.8 mg, 0.12 mmol), dry pyridine (6 drops) and (*S*)-MTPACl (0.24 mmol, 0.45 mL of a 0.53M solution in dry CH_2Cl_2) in dry CH_2Cl_2 (2 mL) was stirred under argon at RT for 18 h. Water was added after it was concentrated under reduced pressure. The mixture was extracted three times with CH_2Cl_2 and the combined organic layers were washed with HCl (2M) and a saturated solution of $NaHCO_3$, dried ($MgSO_4$) and concentrated under reduced pressure. The residue was purified by flash chromatography (hexane:EtOAc = 5:1, R_f = 0.44) to yield the (*R*)-Mosher amide **17**·MTPA-(*R*) (23.5 mg, 61%) as a colorless solid. ^{1}H NMR (400.27 MHz, $CDCl_3$): δ 7.55-7.49 (m, 2H), 7.41-7.36 (m, 3H), 7.35-7.23 (m, 5H), 7.08 (br. s, 1H), 4.51 (AB part of ABX-system, J_{AB} = 14.8 Hz, J_{AX} = 6.0 Hz, J_{BX} = 8.8 Hz, 2H), 3.37 (q, J = 1.5 Hz, 3H).

Similarly, (*R*)-phenyl-[D_1]methylamine [(*R*)-[D_1]**17**, 0.012 g, 0.11 mmol] was converted to the (*R*)-Mosher amide (*R*)-[D_1]**17**·MTPA-(*R*) (0.023 g, 65%, de > 69%). ^{1}H NMR (400.27 MHz, $CDCl_3$): δ 7.55-7.49 (m, 2H), 7.41-7.36 (m, 3H), 7.35-7.23 (m, 5H), 7.08 (br. s, 1H), 4.52 (td, J = 5.8, 2.0 Hz, 0.83H, NCHD), 4.46 (td, J = ~5.8, ~2 Hz, 0.15H, NCHD), 3.37 (q, J = 1.4 Hz, 3H).

Similarly, (*S*)-phenyl-[D_1]methylamine [(*S*)-[D_1]**17**, 0.013 g, 0.12 mmol] was converted to the (*R*)-Mosher amide (*S*)-[D_1]**17**·MTPA-(*R*) (0.005 g, 13%, de > 52%). ^{1}H NMR (400.27 MHz, $CDCl_3$): δ 7.55-7.49 (m, 2H), 7.41-7.36 (m, 3H), 7.35-7.23 (m, 5H), 7.08 (br. s, 1H), 4.52 (td, J = 5.8, 2.0 Hz, 0.24H, NCHD), 4.46 (td, J = ~5.8, ~2 Hz, 0.76H, NCHD), 3.37 (q, J = 1.4 Hz, 3H).

Similarly, (*R*)-phenyl-[D_1]methylamine [(*R*)-[D_1]**17**, 0.011 g, 0.10 mmol] obtained by deblocking authentic sample of (*R*)-*N*-(phenyl-[D_1]methyl)phthalimide was converted to the (*R*)-Mosher amide (*R*)-[D_1]**17**·MTPA-(*R*) (0.005 g, 15%, de > 99%). ^{1}H NMR (400.27 MHz, $CDCl_3$): δ 7.55-7.49 (m, 2H), 7.41-7.36 (m, 3H), 7.35-7.23 (m, 5H), 7.08 (br. s, 1H), 4.46 (td, J = 5.6, ~2 Hz, 1H, NCHD), 3.37 (q, J = 1.4 Hz, 3H).

6. Suzuki-Myiaura coupling with (dimethylphenylsilylmethyl)boronates

Dimethylphenyl(phenylmethyl)- and (*S*)-dimethyl-phenyl(phenyl-[D_1]methyl)silane [19 and (*S*)-[D_1]19]

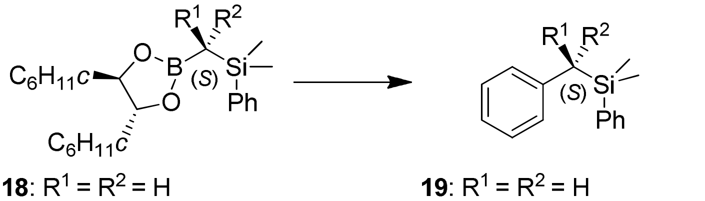

18: $R^1 = R^2 = H$ **19**: $R^1 = R^2 = H$

(*S*)-[D_1]**18**: $R^1 = H, R^2 = D$ (*S*)-[D_1]**19**: $R^1 = H, R^2 = D$

Boronate **18** (0.354 g, 0.91 mmol) and 2M NaOH (3.64 mL) was added to a mixture of bromobenzene (0.429 g, 2.73 mmol, 0.287 mL) and $Pd(PPh_3)_4$ (0.052 g, 0.046 mmol) in dry dioxane (3 mL) under Ar that was stirred for 20 min at RT before heating at 90 °C.[11] After stirring for 18 h, water was added and it was extracted three times with CH_2Cl_2. The combined organic layers were dried (Na_2SO_4) and concentrated under reduced pressure. The crude product was purified by two flash chromatographies (first one: hexane:CH_2Cl_2 = 2:1, R_f = 0.44; second: hexane) to give silane[12] **19** (0.15 g, 59%) as colorless liquid and biphenyl (0.05 g, 15%) as colorless crystals. ^{1}H NMR (400.27 MHz, $CDCl_3$): δ 7.47-7.42 (m, 2H), 7.38-7.30 (m, 3H), 7.19-7.13 (m, 2H), 7.08-7.02 (m, 1H), 6.94-6.90 (m, 2H), 2.30 (s, 2H), 0.24 (s, 6H).

Biphenyl: ^{1}H NMR (400.27 MHz, $CDCl_3$): δ 7.60-7.56 (m, 4H), 7.45-7.40 (m, 4H), 7.36-7.31 (m, 2H); ^{13}C NMR (100.65 MHz, $CDCl_3$): δ 141.3 (2C), 128.7 (4C), 127.24 (2C), 127.16 (4C); HRMS (EI): calcd. for $C_{12}O_{10}$ (M^+) 154.0783, found 154.0781.

Similarly, (*S*)-dimethylphenyl(phenyl-[D_1]methyl)boronate [(*S*)-[D_1]**18**, 0.88 g, 2.27 mmol] was converted to (*S*)-dimethylphenyl(phenyl-[D_1]methyl)silane [(*S*)-[D_1]**19**, 0.28 g, 54%]. ^{1}H NMR (400.27 MHz, $CDCl_3$): δ 7.49-7.44 (m, 2H), 7.40-7.31 (m, 3H), 7.21-7.15 (m, 2H), 7.09-7.04 (m, 1H), 6.96-6.92 (m, 2H), 2.29 (broadened s, 1H, CHD), 0.25 (s, 6H); ^{13}C NMR (100.65 MHz, $CDCl_3$): δ 139.6, 138.5, 133.7 (2C), 129.0, 128.3 (2C), 128.1 (2C), 127.7 (2C), 124.1, 25.8 (t, J = 18.3 Hz, CHD), –3.5 (2C).

Fluorodimethyl(phenylmethyl)- and (*S*)-fluorodimethyl(phenyl-[D_1]methyl)silane [20 and (*S*)-[D_1]20]

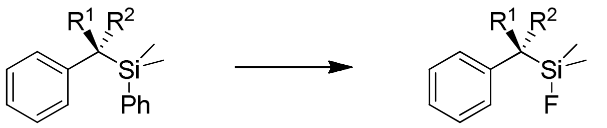

19: $R^1 = R^2 = H$ **20**: $R^1 = R^2 = H$
(*S*)-[D_1]**19**: $R^1 = H, R^2 = D$ (*S*)-[D_1]**20**: $R^1 = H, R^2 = D$

$HBF_4 \cdot Et_2O$ (0.72 g, 4.4 mmol, 0.60 mL) was added to a solution of silane **19** (0.45 g, 2 mmol) in dry CH_2Cl_2 (11 mL) at 0 °C.[13] After stirring for 1 h at 0 °C and 1 h at RT water was added. The mixture was extracted three times with CH_2Cl_2. The combined organic layers were washed with brine, dried ($MgSO_4$) and concentrated under reduced pressure. The crude fluorosilane **20** (0.32 g, 95%) was immediately used in the following reaction. ^{1}H NMR (400.27 MHz, $CDCl_3$): δ 7.15-7.10 (m, 2H), 7.02-6.94 (m, 3H), 2.14 (d, J_{HF} = 5.8 Hz, 2H), 0.08 (d, J_{HF} = 7.4 Hz, 6H).

Similarly, (*S*)-dimethylphenyl(phenyl-[D_1]methyl)silane [(*S*)-[D_1]**19**, 0.30 g, 1.3 mmol] was converted to (*S*)-fluorodimethyl(phenyl-[D_1]methyl)silane [(*S*)-[D_1]**20**, 0.20 g, 92%]. ^{1}H NMR (400.27 MHz, $CDCl_3$): δ 7.14-7.09 (m, 2H), 7.02-6.93 (m, 3H), 2.12 (td, J_{HF} = 5.9 Hz, J_{HD} = 1.9 H, 1H, CHD), 0.08 (d, J = 7.4 Hz, 6H).

Phenyl- and (*S*)-phenyl-[D$_1$]methanol [4 and (*S*)-[D$_1$]4]

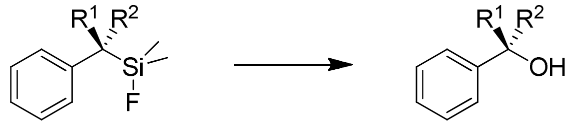

20: $R^1 = R^2 = H$
(*S*)-[D_1]**20**: $R^1 = H, R^2 = D$

4: $R^1 = R^2 = H$
(*S*)-[D_1]**4**: $R^1 = H, R^2 = D$

A mixture of fluorosilane **20** (0.36 g, 2.1 mmol), KF (0.37 g, 6.3 mmol), $NaHCO_3$ (0.18 g, 2.1 mmol) and 30% H_2O_2 (21 mmol, 2.44 mL) in dry methanol (8 mL) and dry THF (8 mL) was refluxed for 2 h.[14] A saturated aqueous solution of $NaHCO_3$ was added and it was extracted three times with Et_2O. The combined organic layers were dried ($MgSO_4$) and concentrated under reduced pressure. The crude was purified by flash chromatography (hexane:EtOAc = 5:1, R_f= 0.22) to give phenylmethanol (**4**, 0.15 g, 67%) as a colorless liquid. ^{1}H NMR (400.27 MHz, $CDCl_3$): δ 7.36-7.34 (m, 4H), 7.31-7.26 (m, 1H), 4.68 (s, 2H), 1.69 (broad s, 1H).

Similarly, (*S*)-fluorodimethyl(phenyl-[D_1]methyl)silane [(*S*)-[D_1]**20**, 0.20 g, 1.19 mmol] was converted to (*S*)-phenyl-[D_1]methanol [(*S*)-[D_1]**4**, 0.057 g, 44%]. ^{1}H NMR (400.27 MHz, $CDCl_3$): δ 7.36-7.34 (m, 4H), 7.32-7.26 (m, 1H), 4.66 (t, J_{HD} = 1.9 H, 1H, CHD), 1.68 (broad s, 1H); ^{13}C NMR (100.65 MHz, $CDCl_3$): δ 140.8, 128.6 (2C), 127.7, 127.0 (2C), 65.0 (t, J = 21.7 Hz, CHD). This alcohol was converted to the (*R*)-Mosher ester by the method used for the esterification of (*R*)-[D_1]**4** as given above (see 2. Stille coupling with tributylstannylmethanols). The ^{1}H NMR spectrum (400.27 MHz) was identical to that of a sample prepared from authentic (*S*)-[D_1]**4** (see 2. Stille coupling with tributylstannylmethanols).

7. Spectra

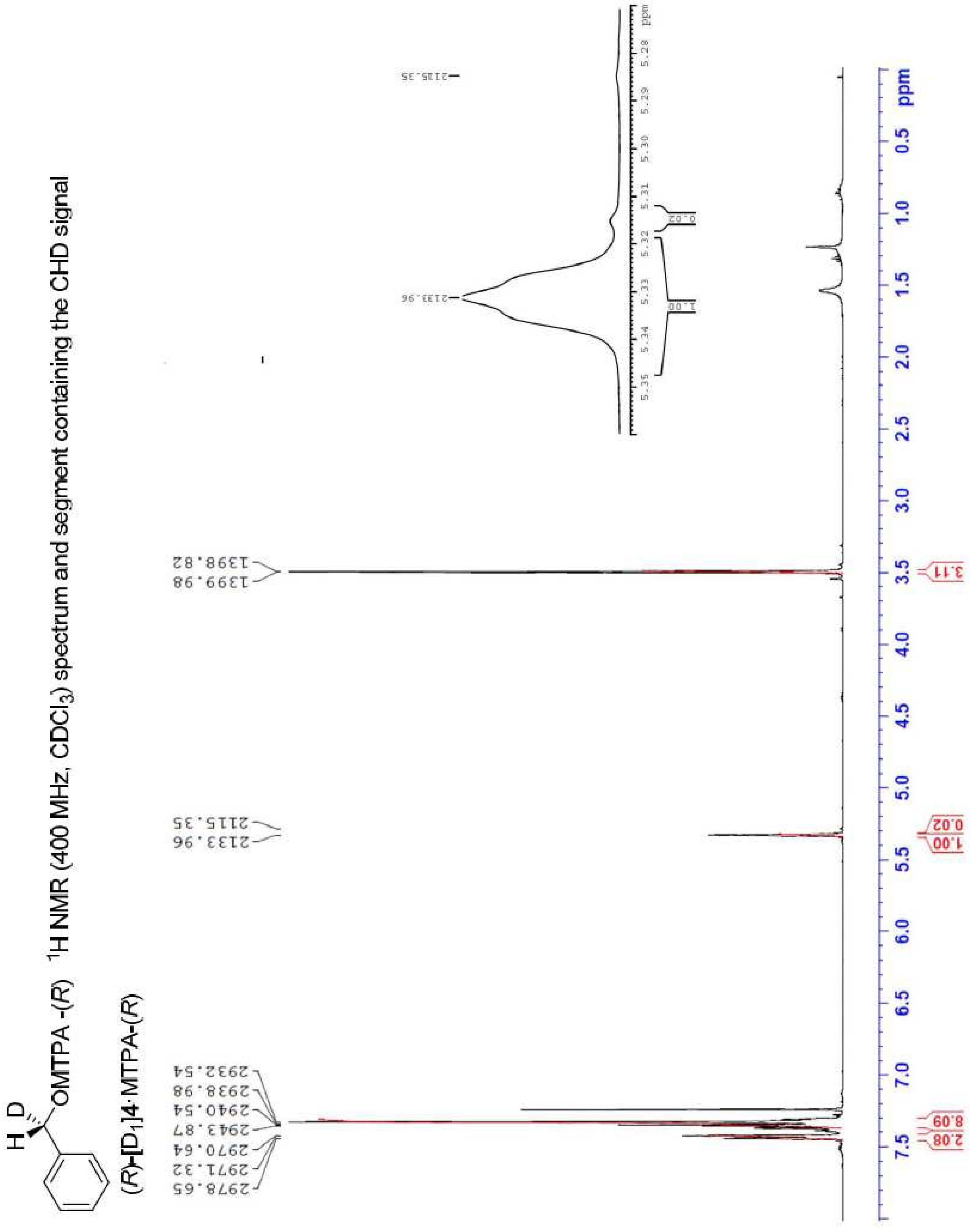

OMTPA -(R)
(R)-[D1]4·MTPA-(R)
1H NMR (400 MHz, CDCl3) spectrum and segment containing the CHD signal
2978.65
2971.32
2970.64
2943.87
2940.54
2938.98
2932.54
2133.96
2115.35
1399.98
1398.82
ppm

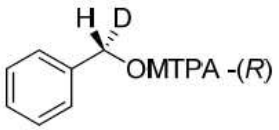

^{1}H NMR (400 MHz, $CDCl_3$) spectrum and the segment containing the CHD signal

(*S*)-[D_1]**4**·MTPA-(*R*)

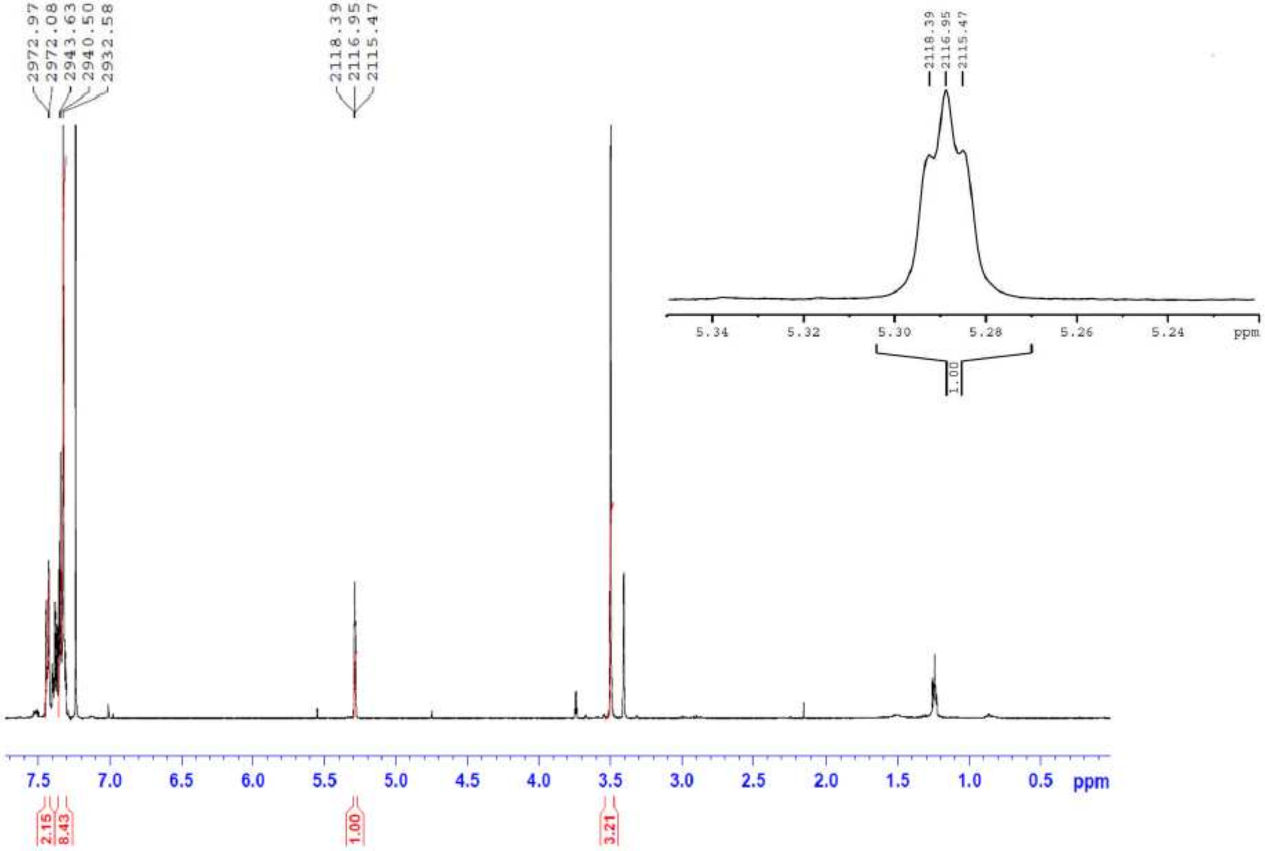

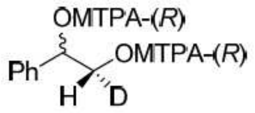

^1H NMR (400 MHz, $CDCl_3$) spectrum and segments containing the CHD signals

(2*R*)-[2-D_1]**7**·MTPA-(*R*)

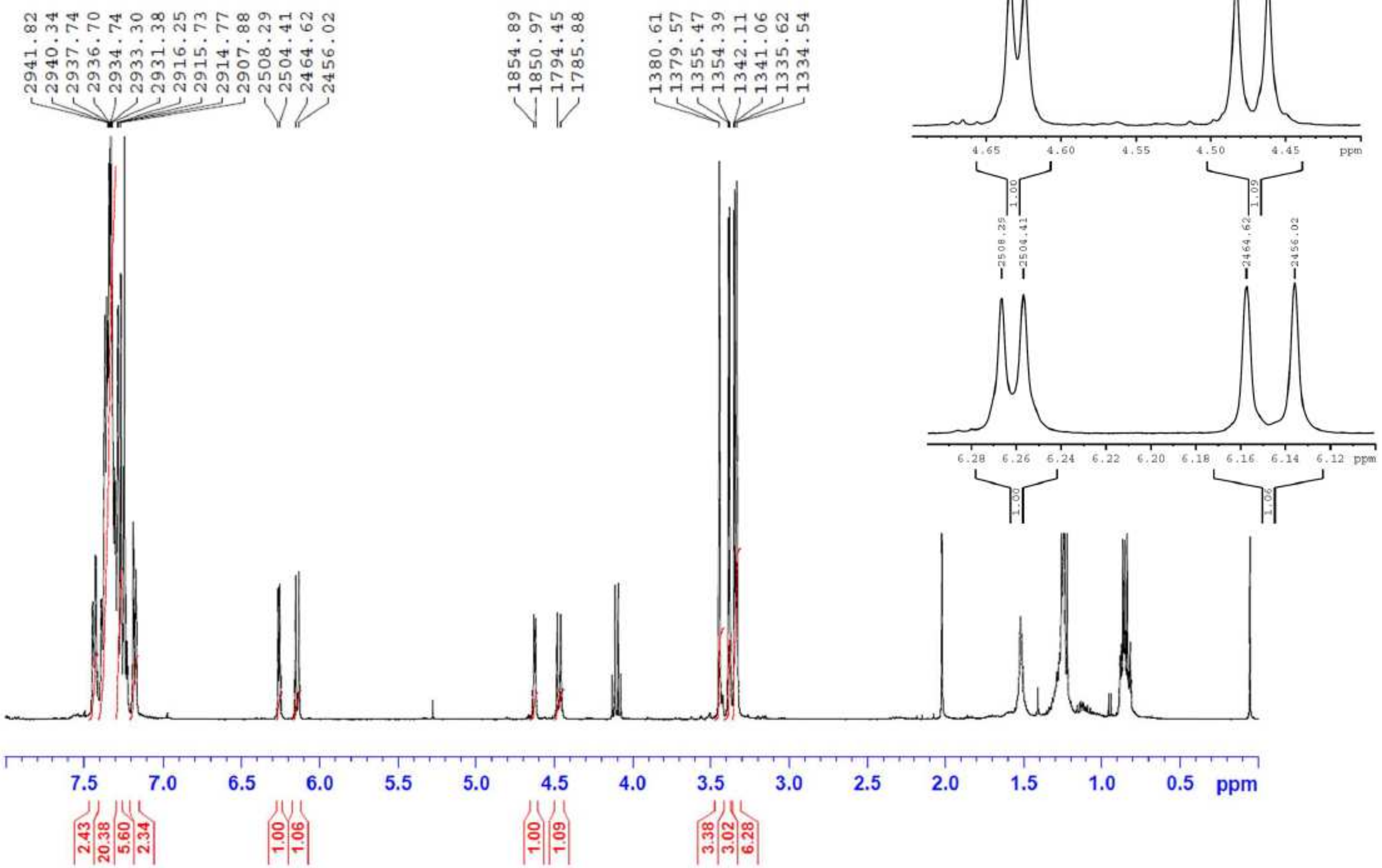

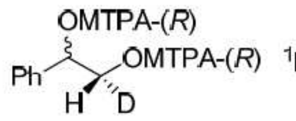

^{1}H NMR (400 MHz, $CDCl_3$) spectrum and segments containing the CHD signals

(2*S*)-[2-D$_1$]**7**·MTPA-(*R*)

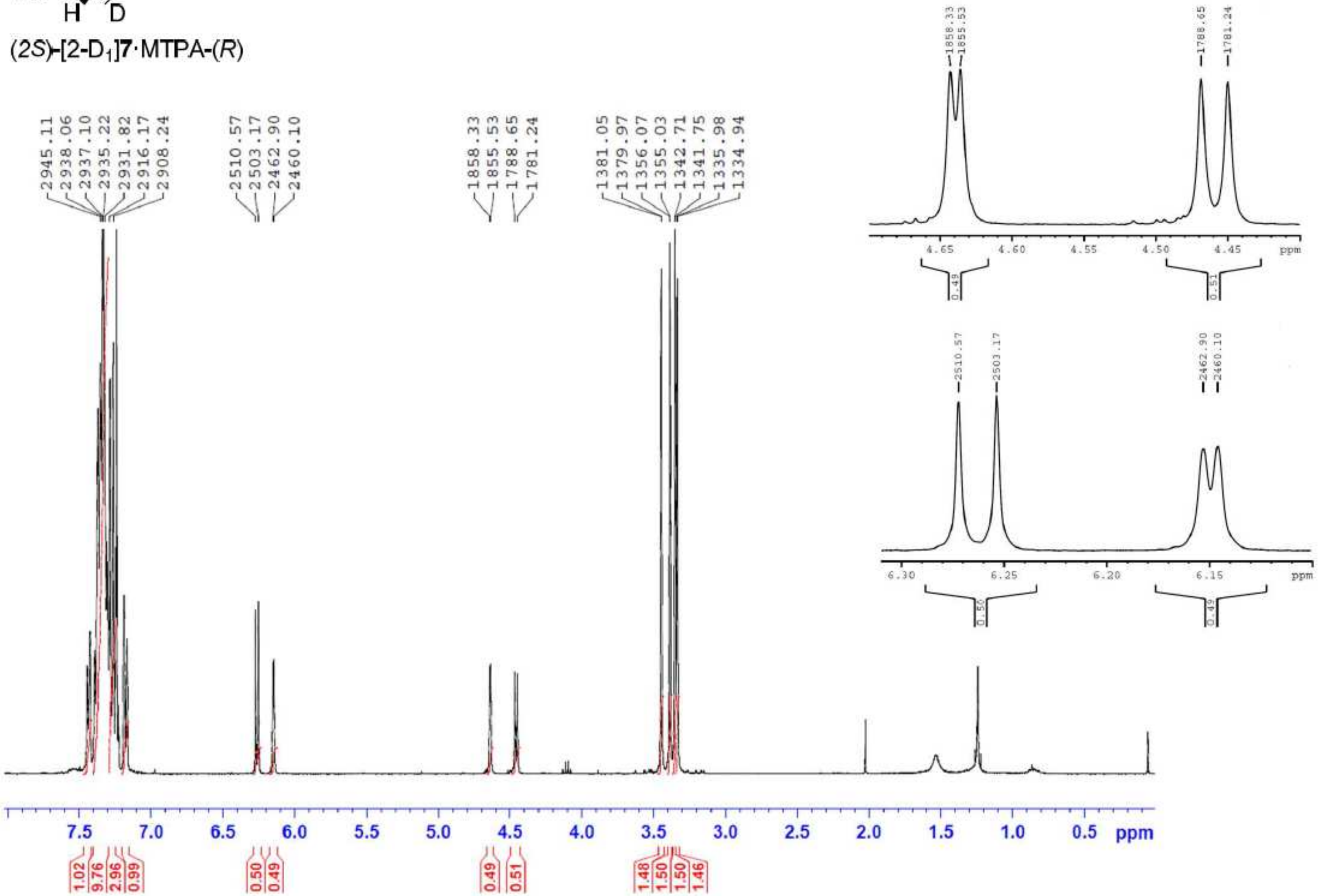

Bu_3SnCH_2SAc

11

^1H NMR (400 MHz, $CDCl_3$) spectrum

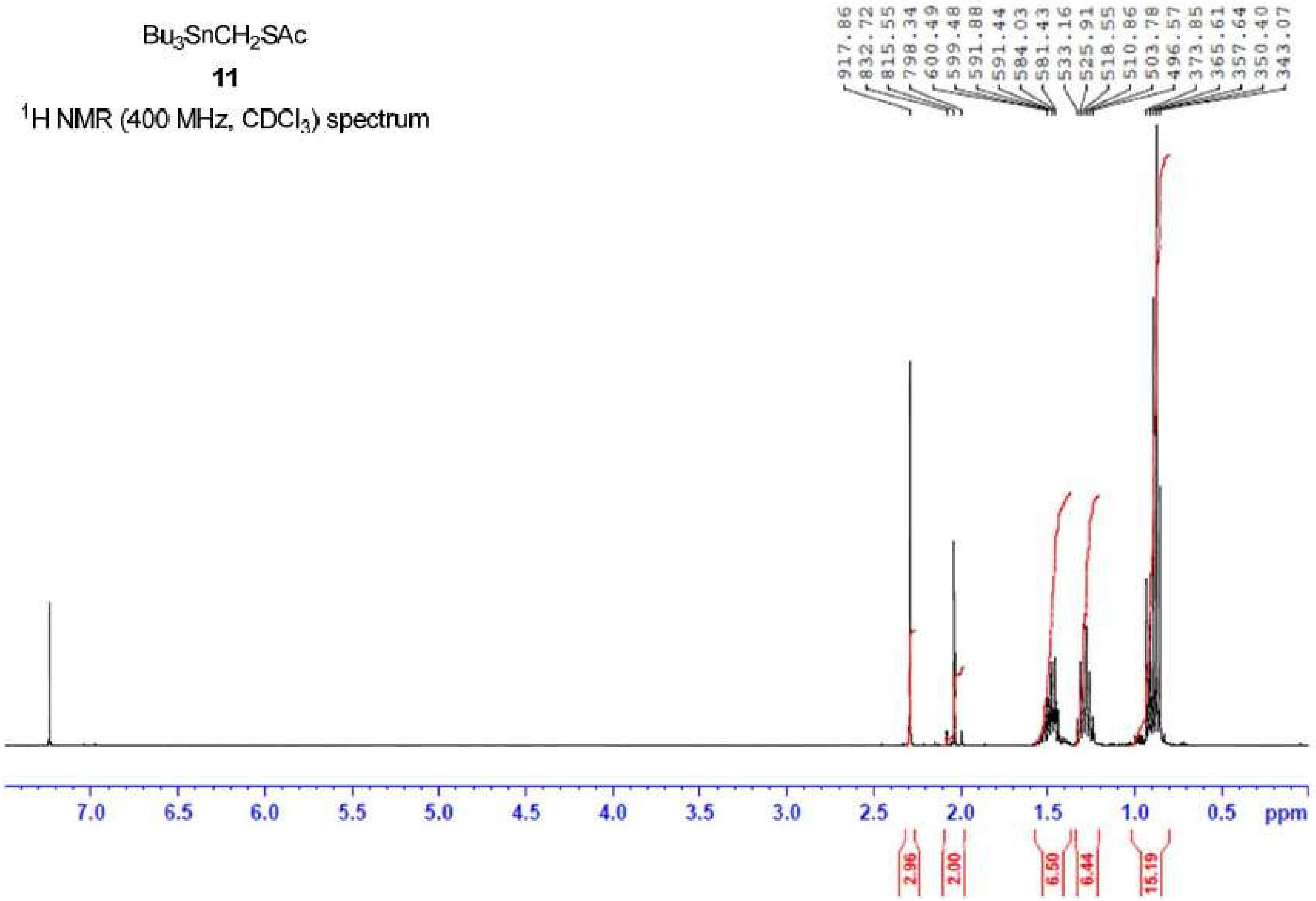

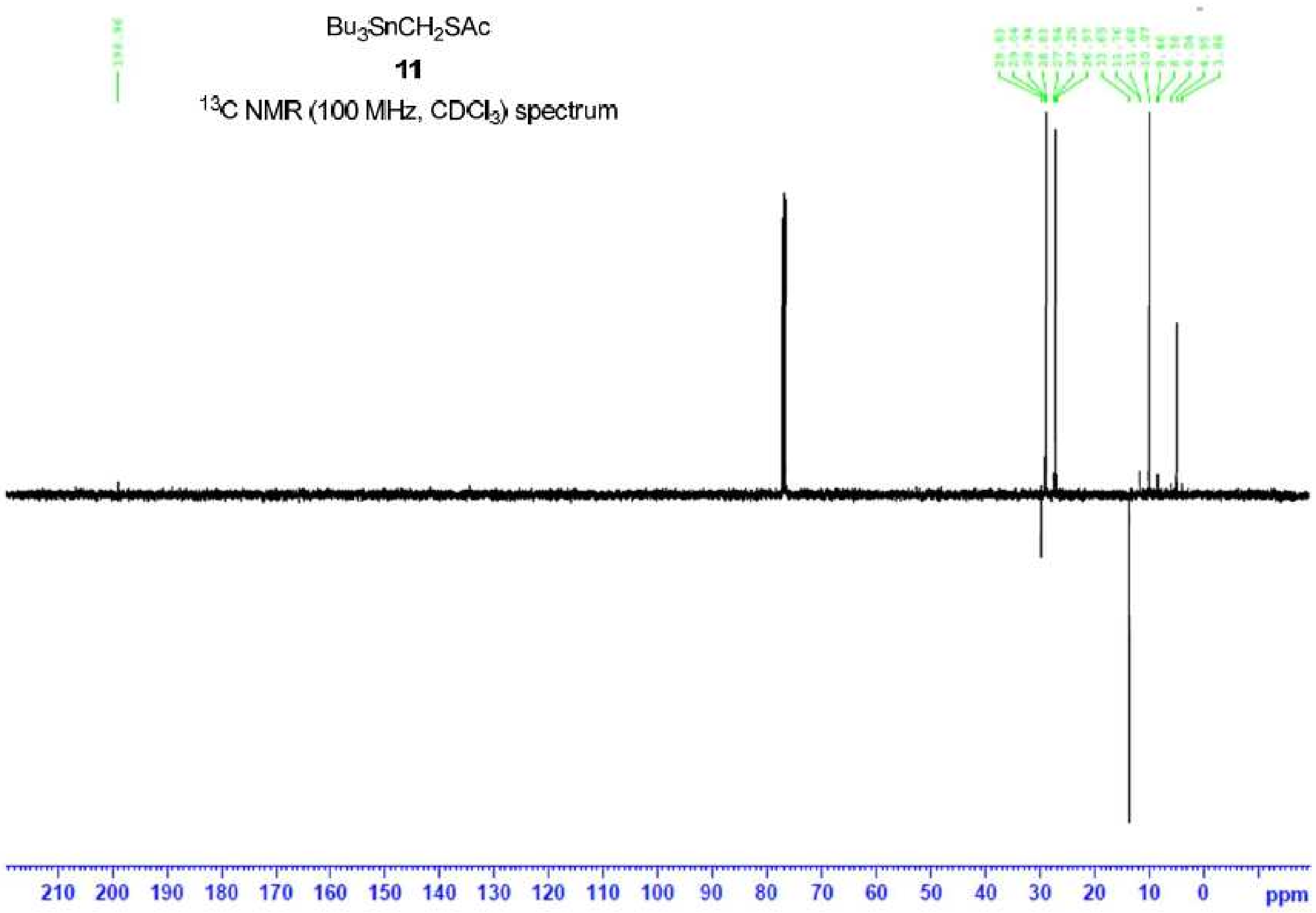

Bu3SnCH2SAc
11
13C NMR (100 MHz, CDCl3) spectrum
210 200 190 180 170 160 150 140 130 120 110 100 90 80 70 60 50 40 30 20 10 0
ppm

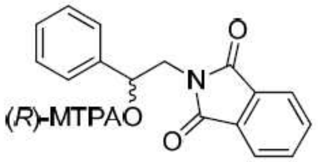

15·MTPA-(*R*)

^{1}H NMR (600 MHz, $CDCl_3$) spectrum and segments containing the CH_2 signals

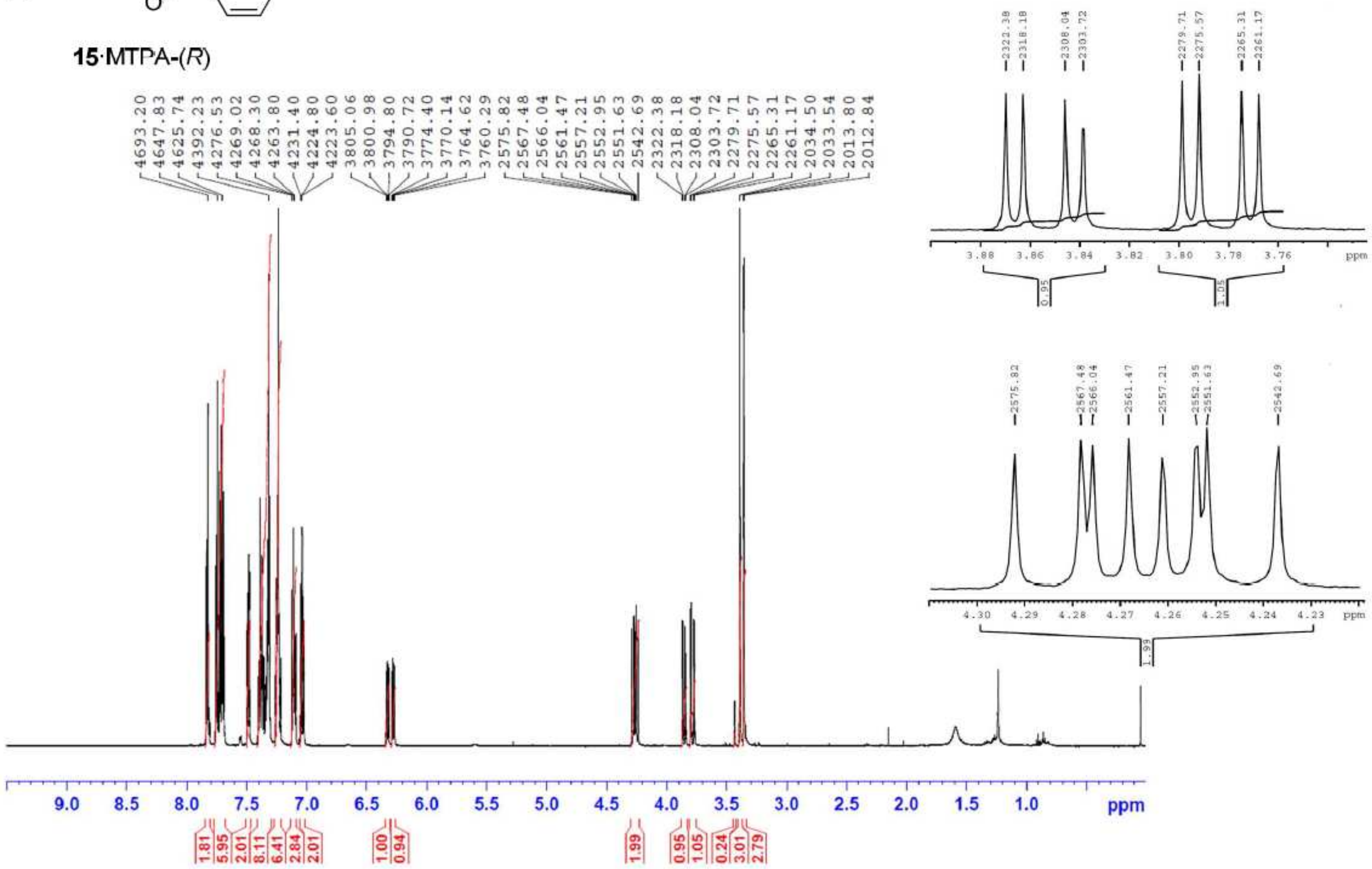

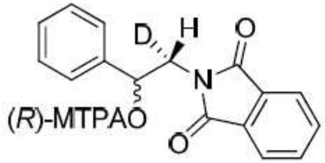

^{1}H NMR (600 MHz, $CDCl_3$) spectrum and segments containing the CHD signals

(2*S*)-[2-D_1]**15**·MTPA-(*R*)

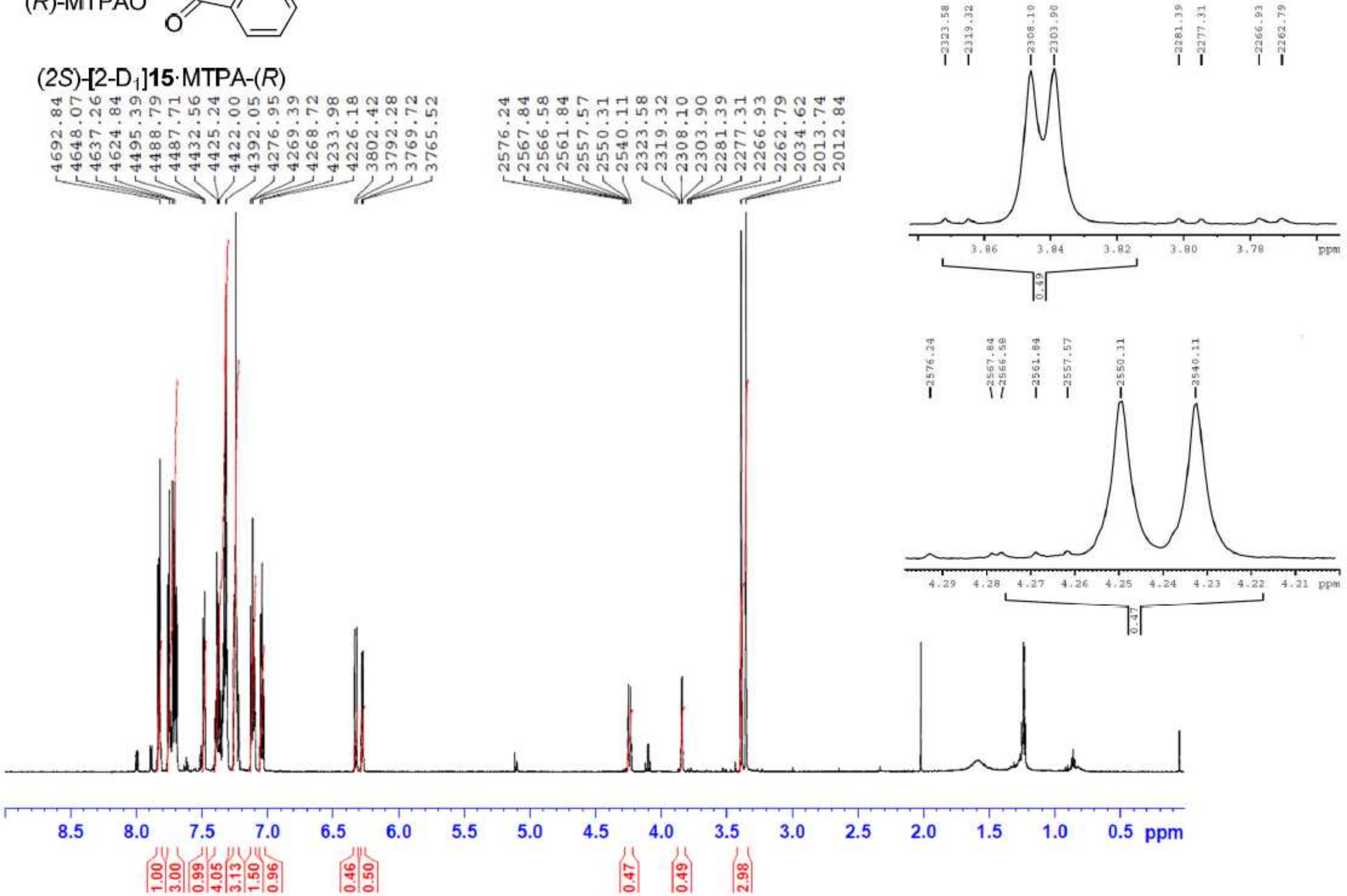

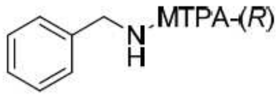

^{1}H NMR (400 MHz, $CDCl_3$) spectrum and segment containing the CH_2 signals

17·MTPA-(*R*)

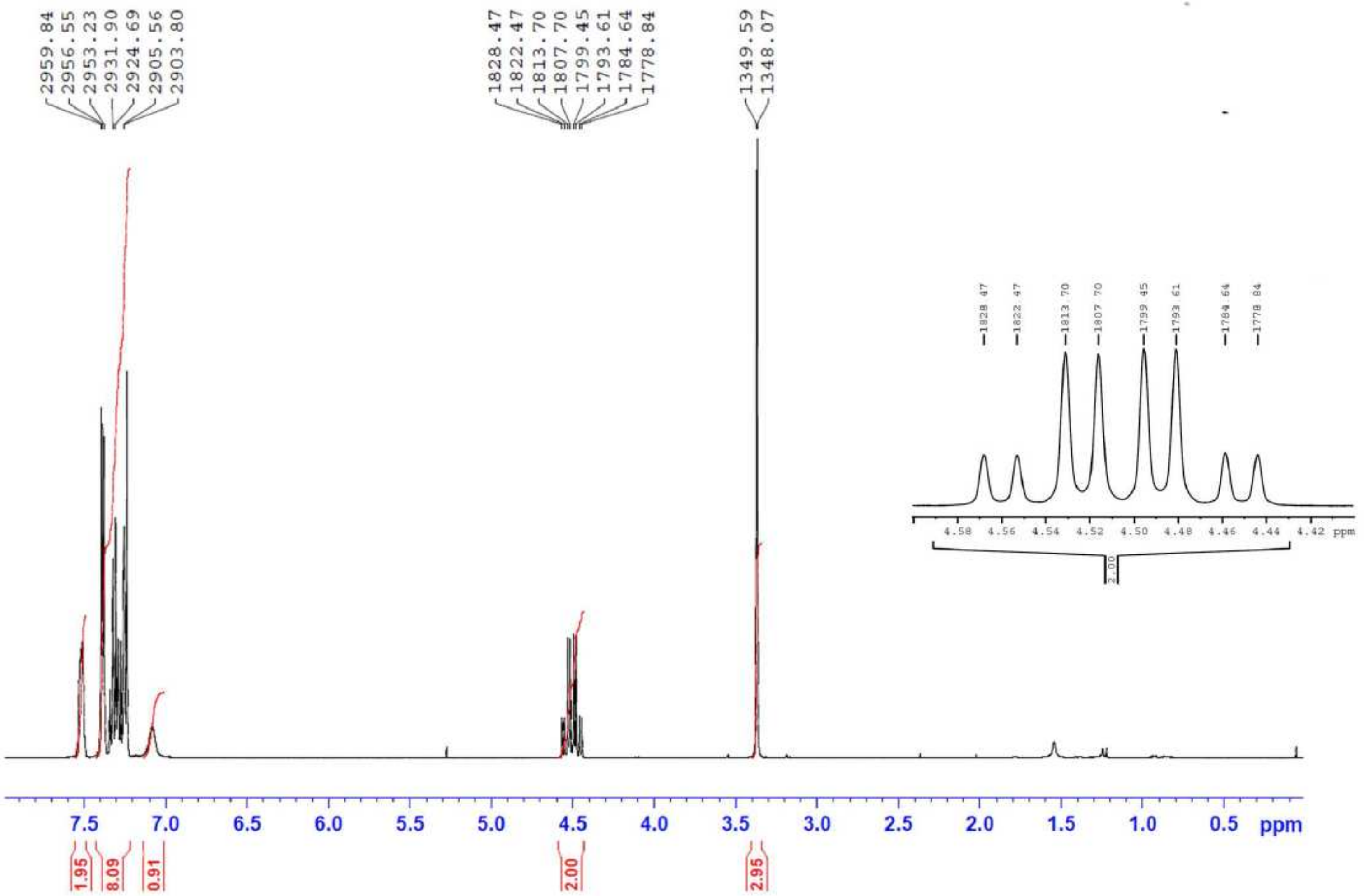

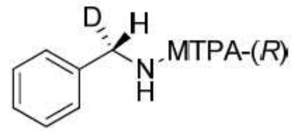

^{1}H NMR (400 MHz, $CDCl_3$) spectrum and segment containing the CHD signal

(*S*)-[D_1]**17**·MTPA-(*R*) (reference sample)

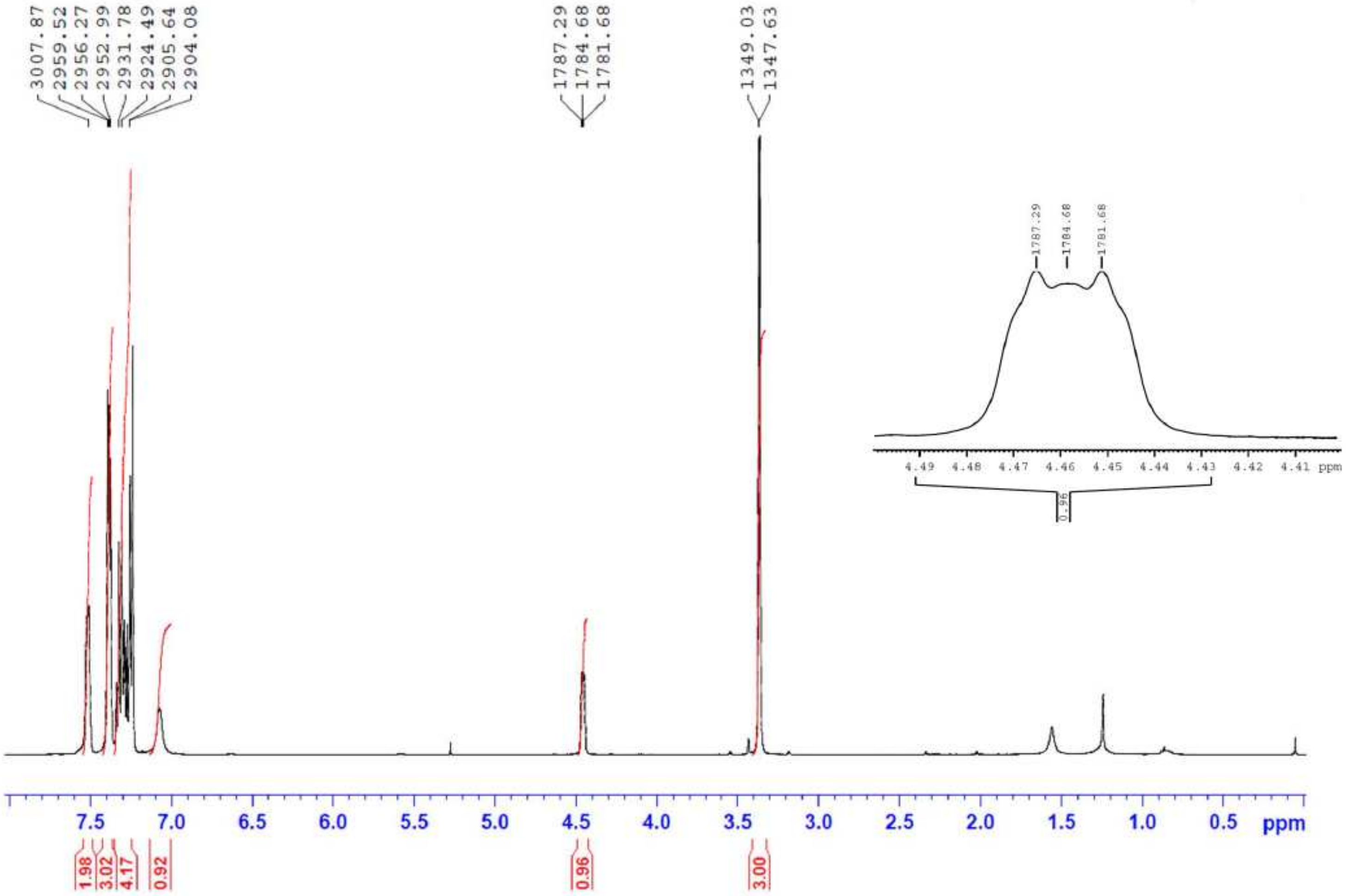

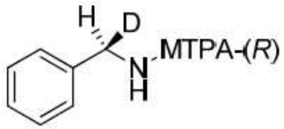

(*R*)-[D$_1$]**17**·MTPA-(*R*)

^{1}H NMR (400 MHz, $CDCl_3$)spectrum and segment containing the CHD signal

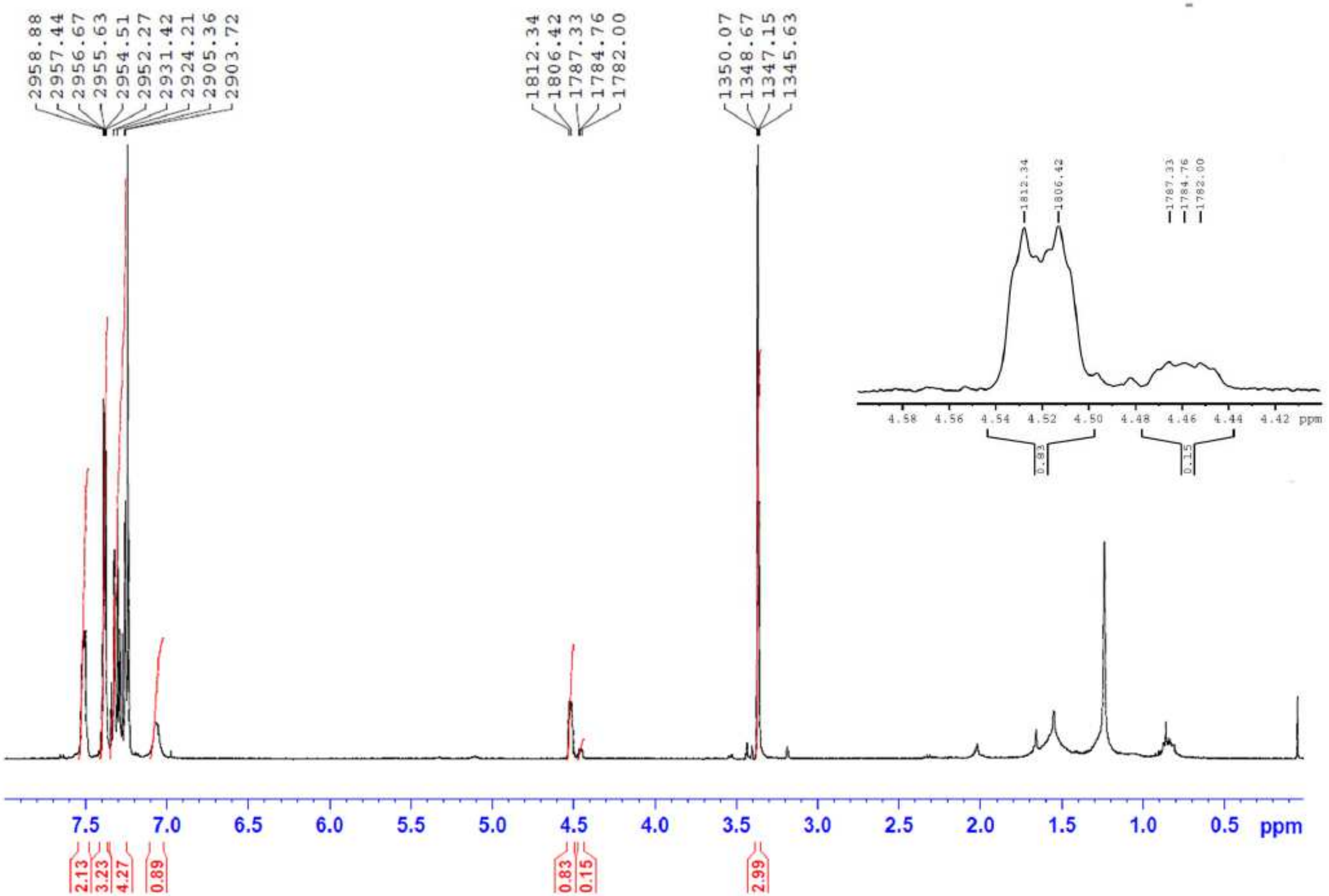

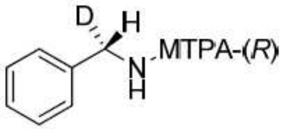

^{1}H NMR (400 MHz, $CDCl_3$) spectrum and segment containing the CHD signals

(*S*)-[D$_1$]**17**·MTPA-(*R*)

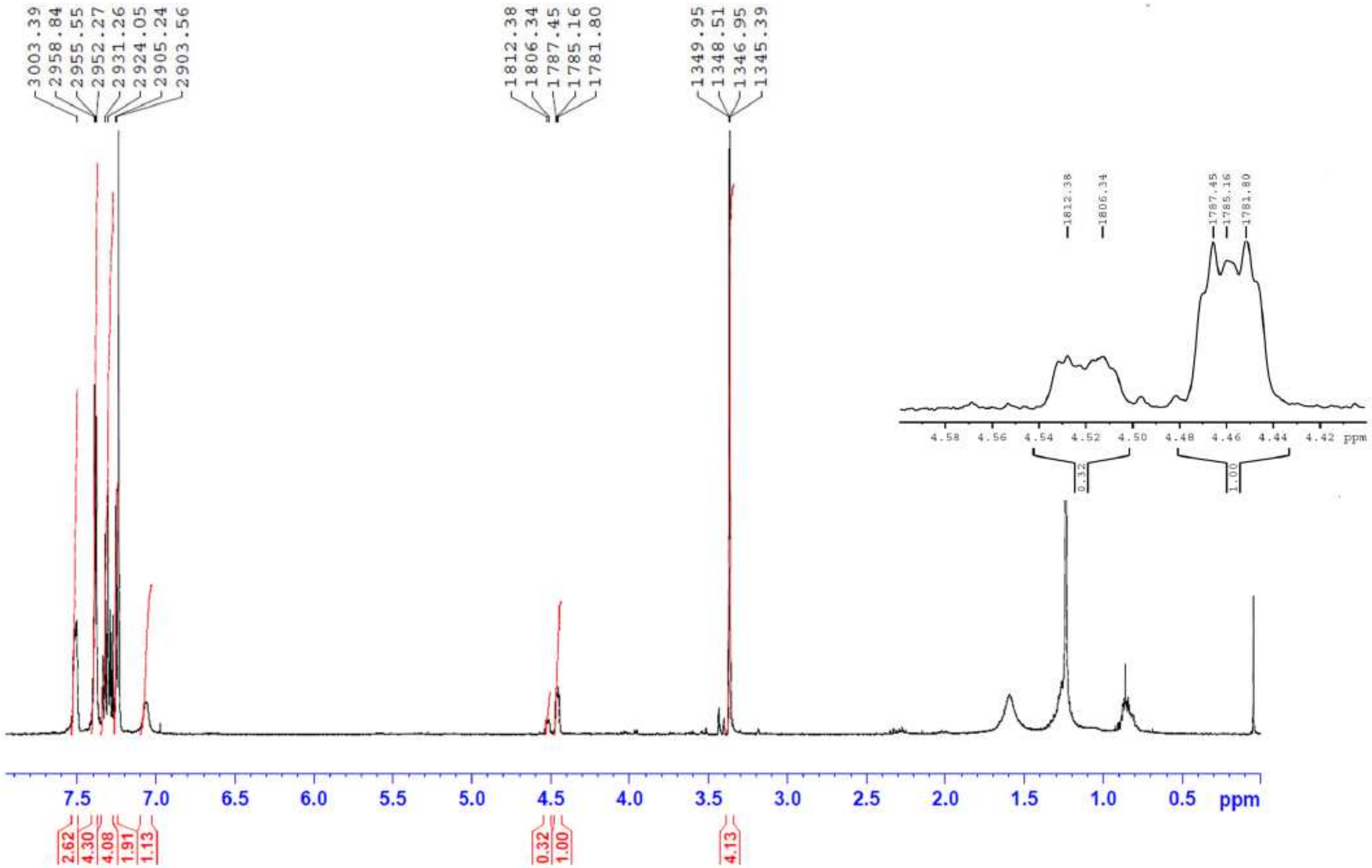

8. Literature

1. M. Kosugi, T. Sumiya, K. Ohhashi, H. Sano, T. Migita, *Chem. Lett.* **1985**, 997-998.
2. J. Ye, R. K. Bhatt, J. R. Falck, *J. Am. Chem. Soc.* **1994**, *116*, 1-5.
3. A. Wieczorek, F. Hammerschmidt, *J. Org. Chem.* **2012**, *77*, 10021-10034.
4. D. C. Kapeller, *Dissertation*, Universität Wien, **2008**.
5. T. Y. S. But, P. H. Toy, *Chem. Asian J.* **2007**, *2*, 1340-1355.
6. S. M. Al-Mousawi, M. A. El-Apasery, N. H. Al-Kanderi, *ARKIVOC* **2008**, *XVI*, 268-278.
7. P. Gupta, B. A. Shah, R. Parshad, G. N. Quazi, S. T. Taneja, *Green Chem.* **2007**, *9*, 1120-1125.
8. A. Terada, *Nippon Kagaku Zasshi* **1956**, *77*, 1265-1267, *Chem. Abstr.* **1959**, *53*, 5185.
9. V. Pace, P. Hoyos, M. Fernández, J.V. Sinisterra, A. R. Alcántara, *Green Chem.* **2010**, *12*, 1380-1382.
10. W. H. Pearson, M. J. Postich, *J. Org. Chem.* **1992**, *57*, 6354-6357.
11. N. Miyaura, K. Yamada, H. Suginome, A. Suzuki, *J. Am. Chem. Soc.* **1985**, *107*, 972-980.
12. J. R. Huckins, S. D. Rychnovsky, *J. Org. Chem.* **2003**, *68*, 10135-10145.
13. I. Fleming, R. Henning, D. C. Parker, H. E. Plaut, P. E. J. Sanderson, *J. Chem. Soc., Perkin Trans. 1* **1995**, 317-337.
14. H.-J. Knölker, G. Wanzl, *Synlett* **1995**, 378-382.
